# Supplementary figures and images for: The human cytomegalovirus-encoded pUS28 antagonizes CD4+ T cell recognition by targeting CIITA
Source: eLife. 2025 Jul 3;14:e96414. doi: 10.7554/eLife.96414 (PMC12226020; doi:10.7554/eLife.96414)

Figure 3A

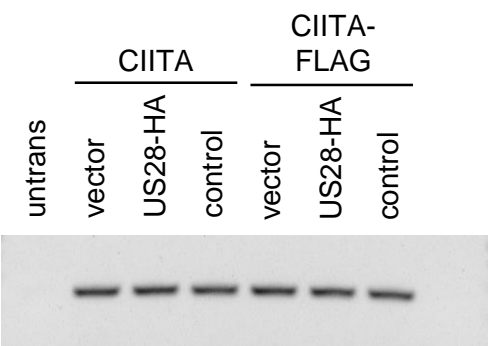

Supplement: Figure 3—source data 1. [file elife-96414-fig3-data1.zip › Figure 3 - source data 1/Fig.3A.pdf]

Figure 3B

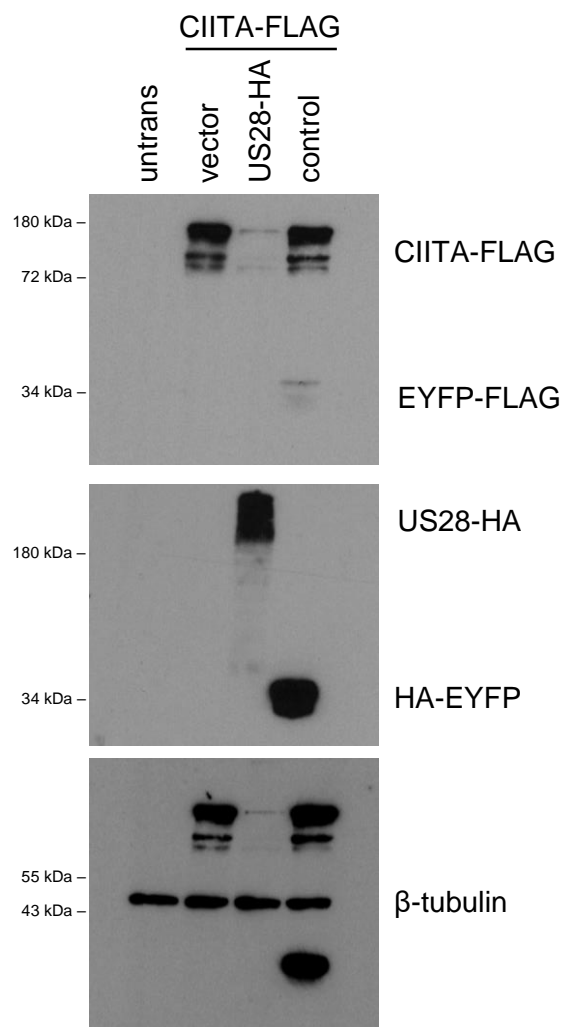

Supplement: Figure 3—source data 1. [file elife-96414-fig3-data1.zip › Figure 3 - source data 1/Fig.3B.pdf]

Figure 3C

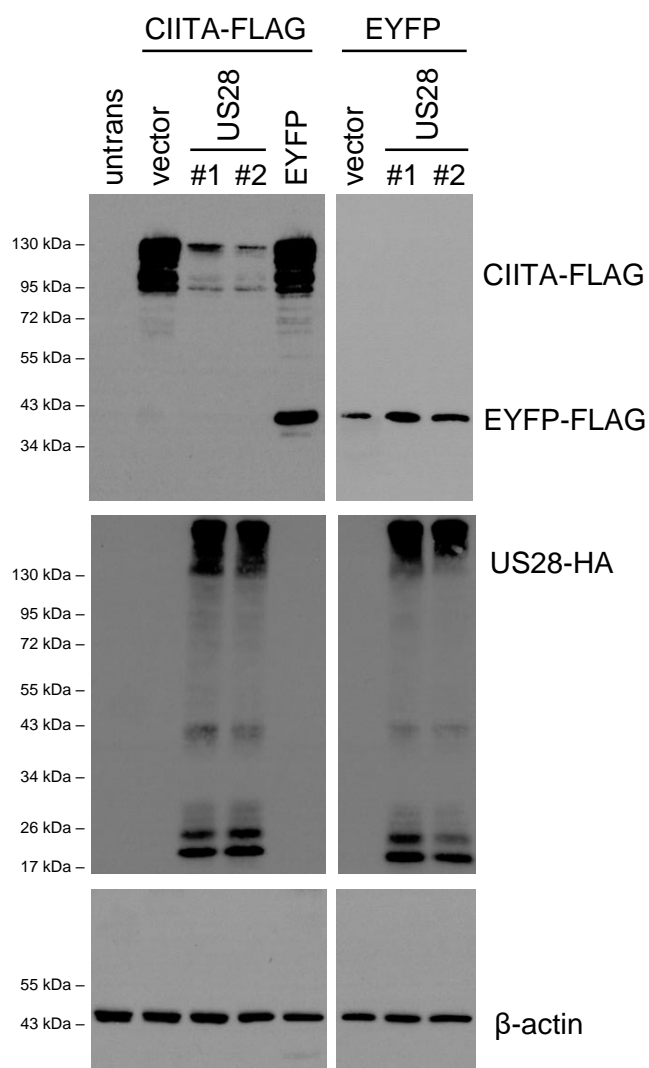

Supplement: Figure 3—source data 1. [file elife-96414-fig3-data1.zip › Figure 3 - source data 1/Fig.3C.pdf]

Figure 3D

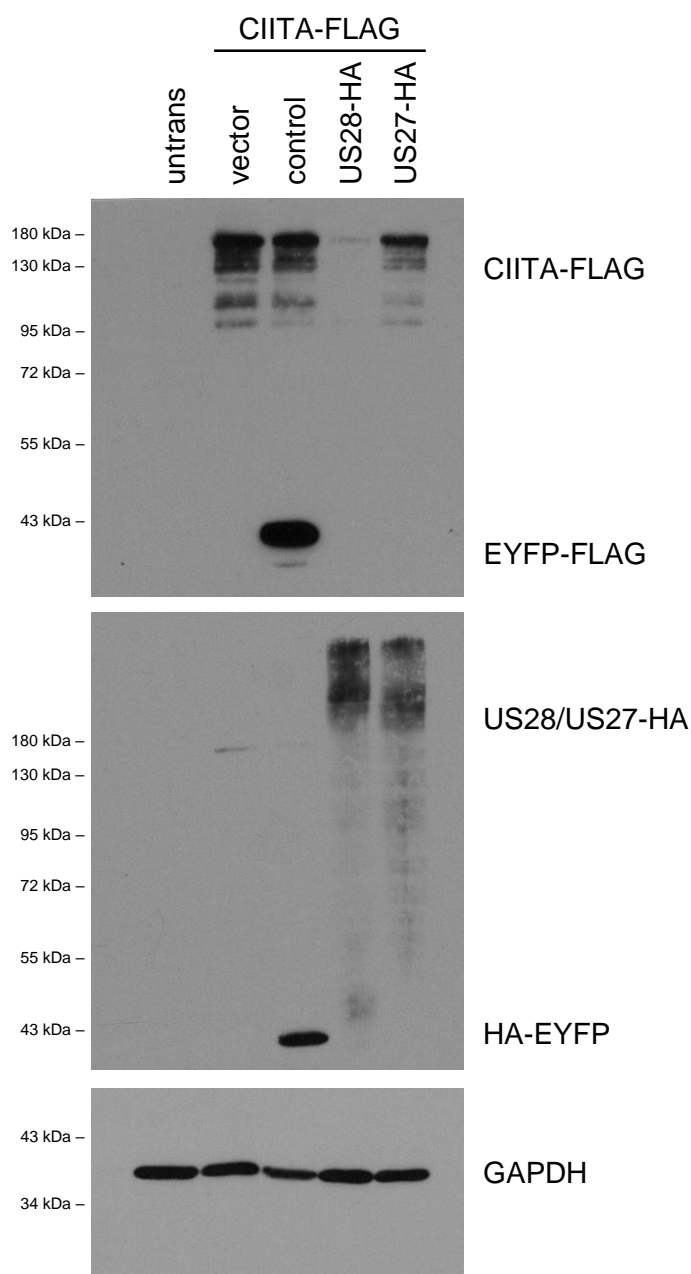

Supplement: Figure 3—source data 1. [file elife-96414-fig3-data1.zip › Figure 3 - source data 1/Fig.3D.pdf]

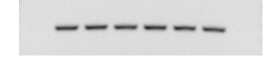

Supplement: Figure 3—source data 2. [file elife-96414-fig3-data2.zip › Figure 3 - source data 2/Fig3A.tif]

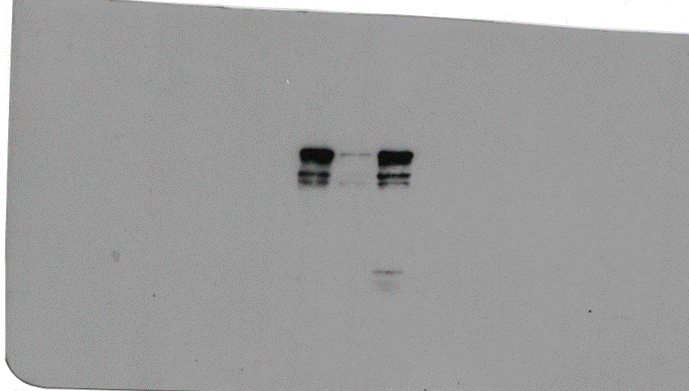

Supplement: Figure 3—source data 2. [file elife-96414-fig3-data2.zip › Figure 3 - source data 2/Fig3B_Flag.tif]

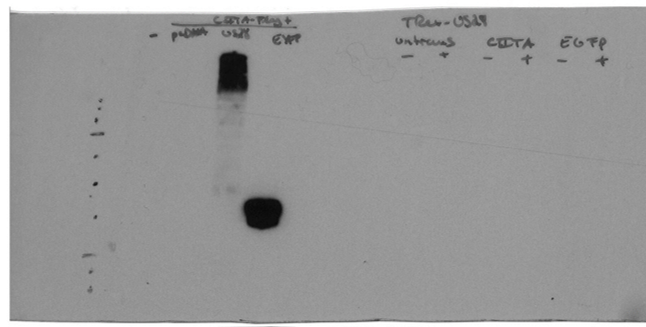

Supplement: Figure 3—source data 2. [file elife-96414-fig3-data2.zip › Figure 3 - source data 2/Fig3B_HA.tif]

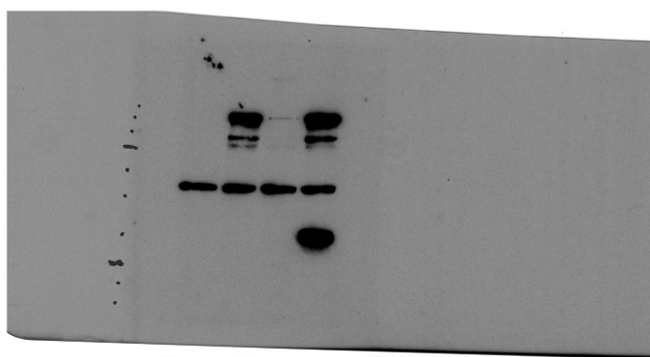

Supplement: Figure 3—source data 2. [file elife-96414-fig3-data2.zip › Figure 3 - source data 2/Fig3B_Tubulin.tif]

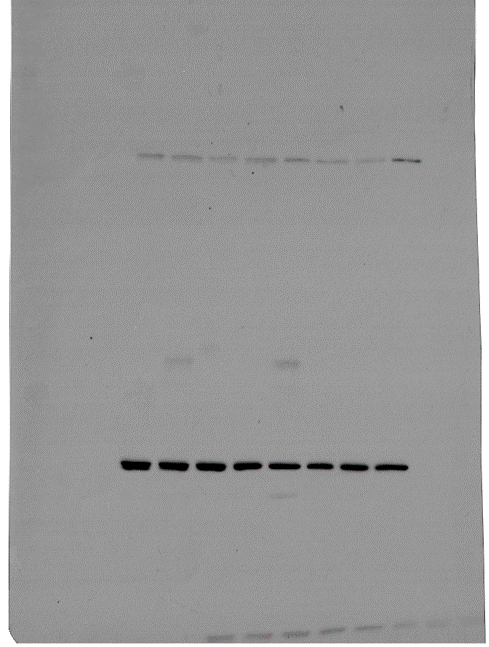

Supplement: Figure 3—source data 2. [file elife-96414-fig3-data2.zip › Figure 3 - source data 2/Fig3C_Actin.tif]

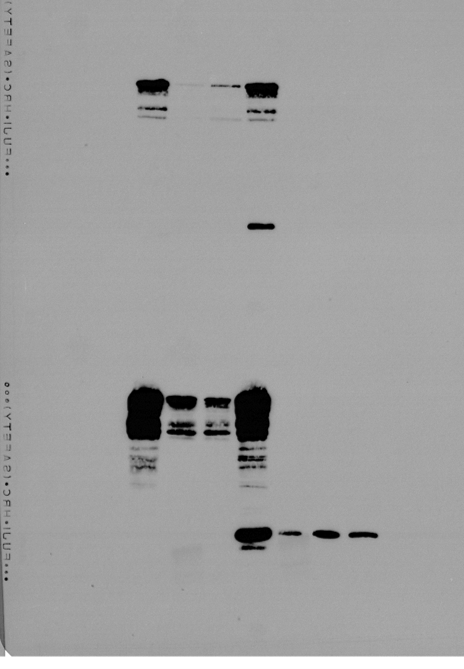

Supplement: Figure 3—source data 2. [file elife-96414-fig3-data2.zip › Figure 3 - source data 2/Fig3C_EYFP-Flag.tif]

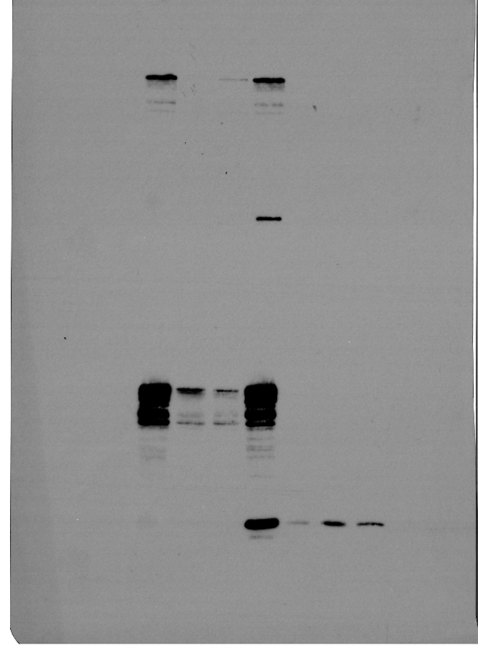

Supplement: Figure 3—source data 2. [file elife-96414-fig3-data2.zip › Figure 3 - source data 2/Fig3C_Flag.tif]

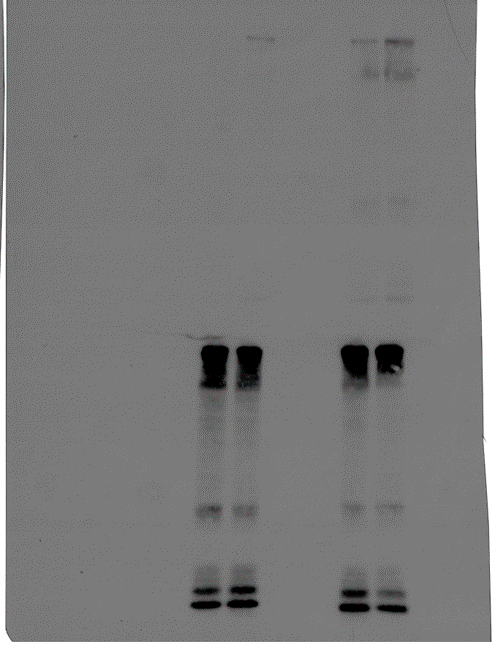

Supplement: Figure 3—source data 2. [file elife-96414-fig3-data2.zip › Figure 3 - source data 2/Fig3C_HA.tif]

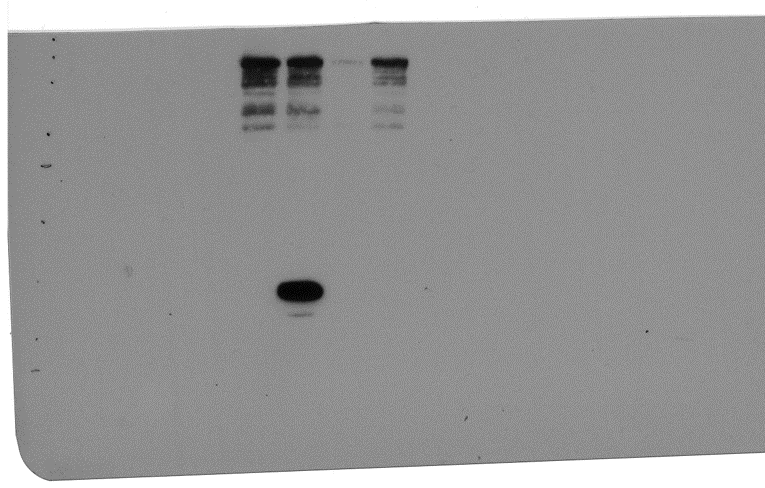

Supplement: Figure 3—source data 2. [file elife-96414-fig3-data2.zip › Figure 3 - source data 2/Fig3D_Flag.tif]

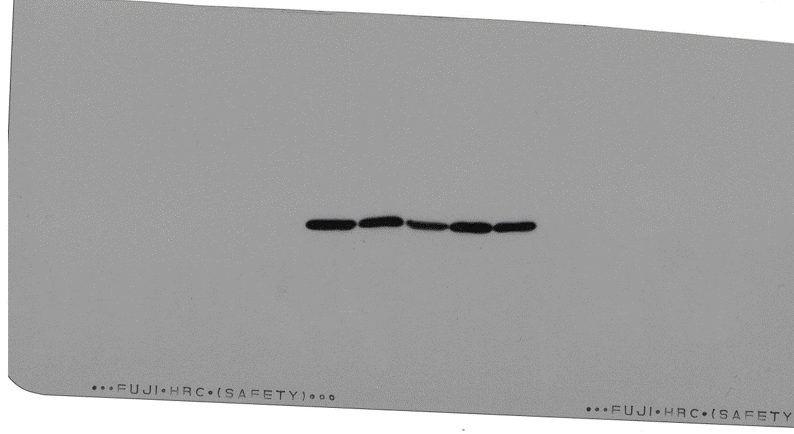

Supplement: Figure 3—source data 2. [file elife-96414-fig3-data2.zip › Figure 3 - source data 2/Fig3D_GAPDH.tif]

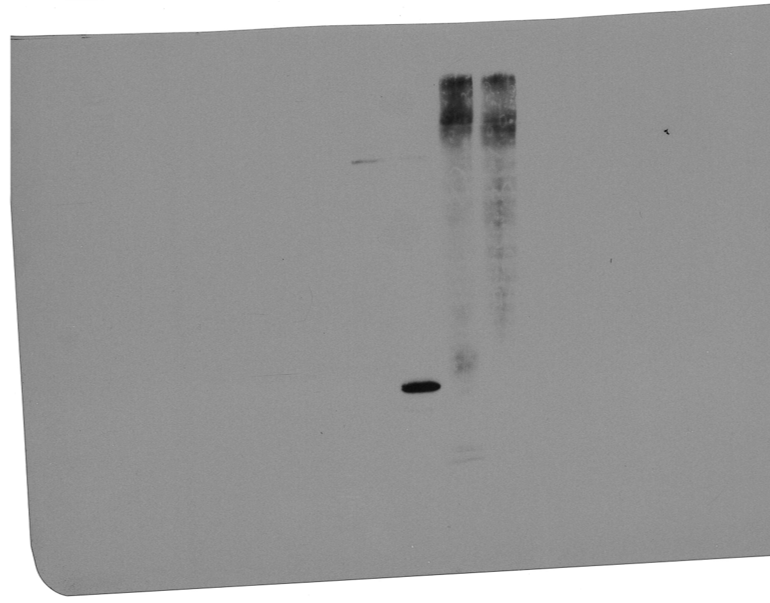

Supplement: Figure 3—source data 2. [file elife-96414-fig3-data2.zip › Figure 3 - source data 2/Fig3D_HA.tif]

Figure 5A

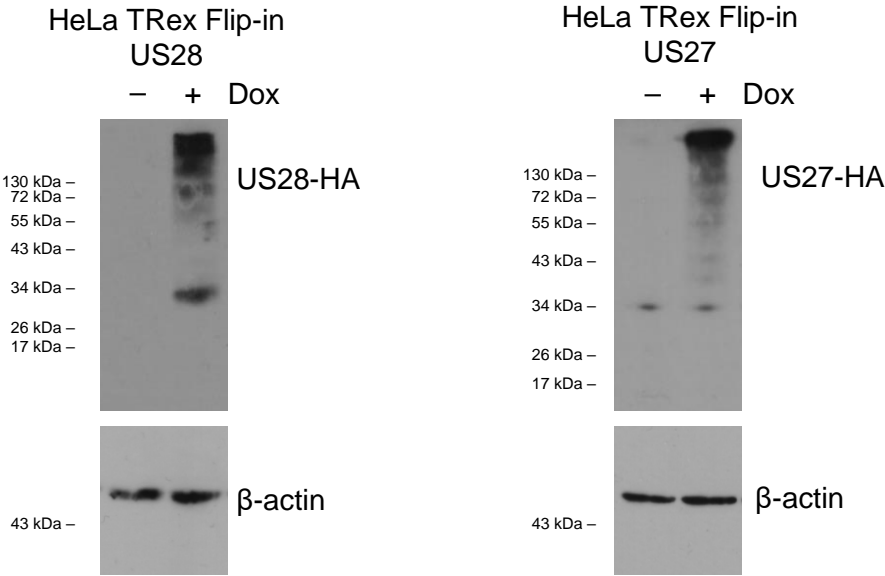

Supplement: Figure 5—source data 1. [file elife-96414-fig5-data1.zip › Figure 5 - source data 1/Fig.5A.pdf]

Figure 5D

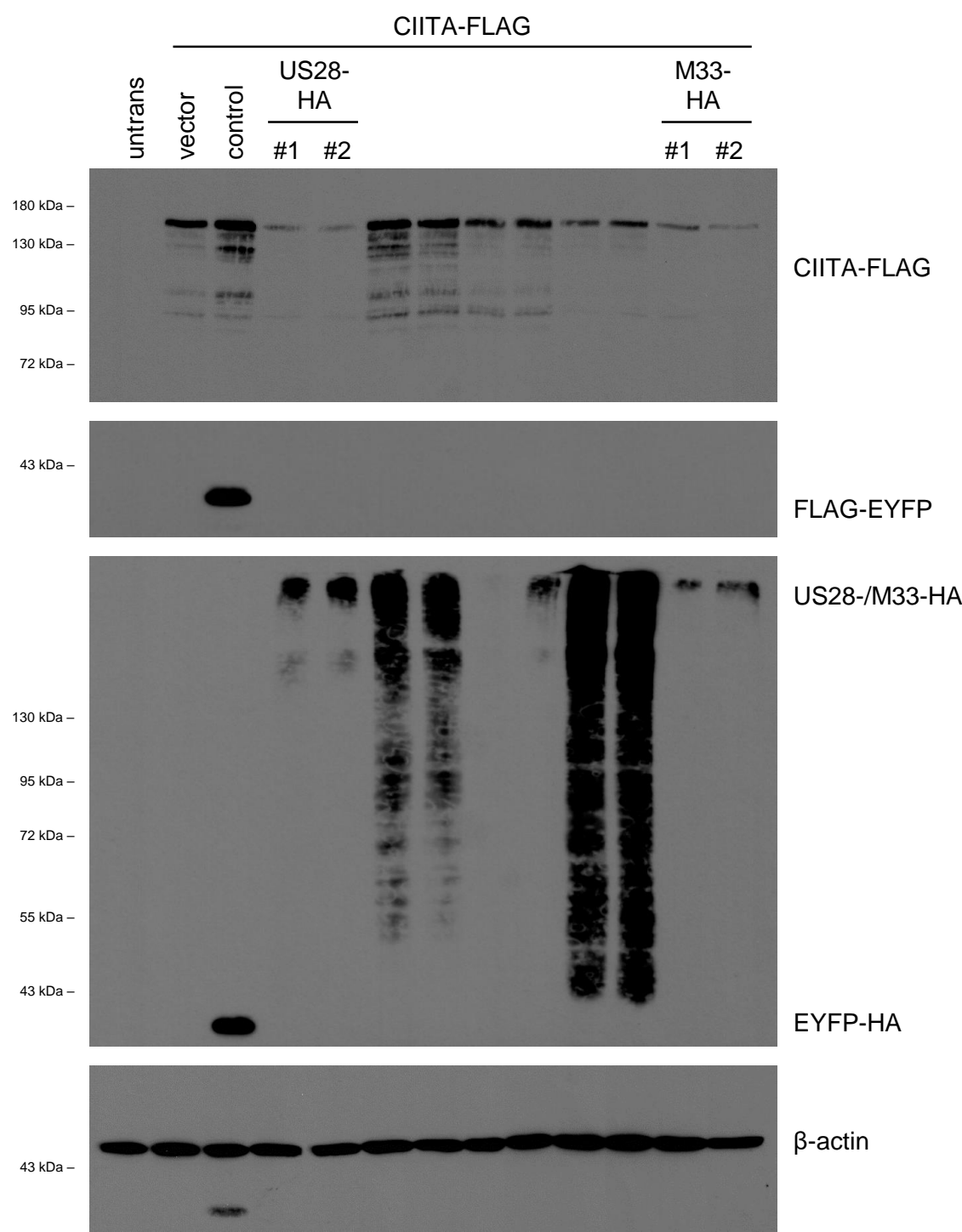

Supplement: Figure 5—source data 1. [file elife-96414-fig5-data1.zip › Figure 5 - source data 1/Fig.5D.pdf]

Figure 5E

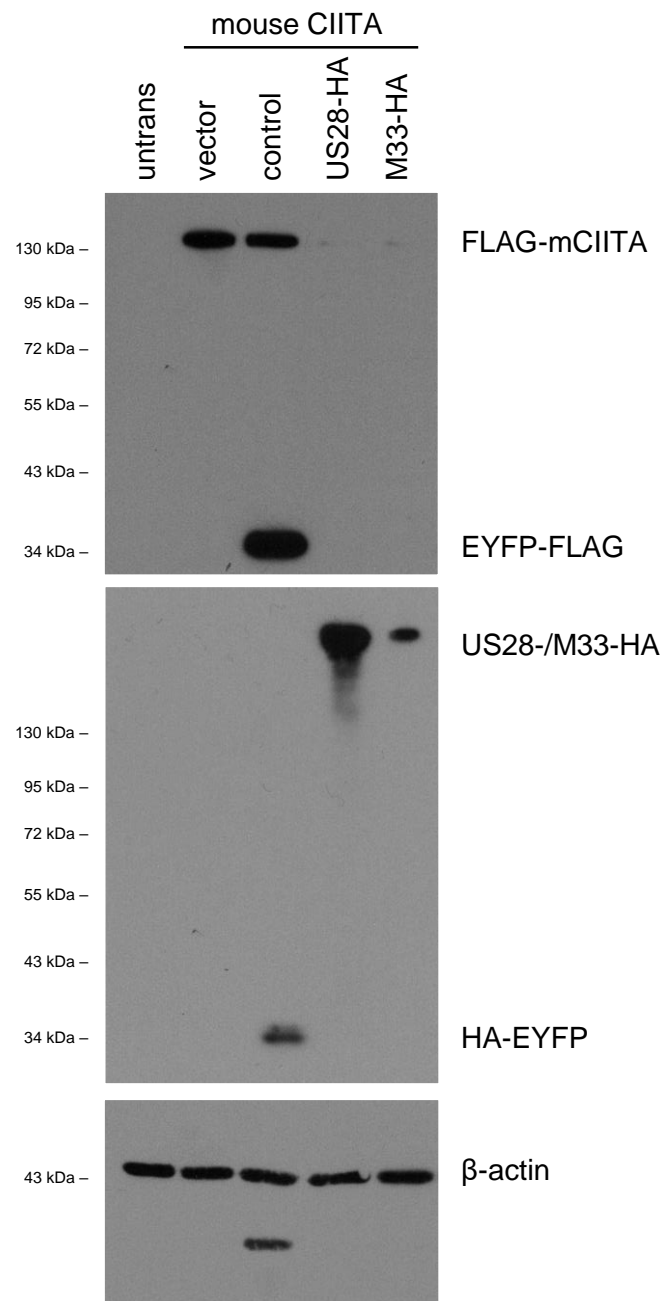

Supplement: Figure 5—source data 1. [file elife-96414-fig5-data1.zip › Figure 5 - source data 1/Fig.5E.pdf]

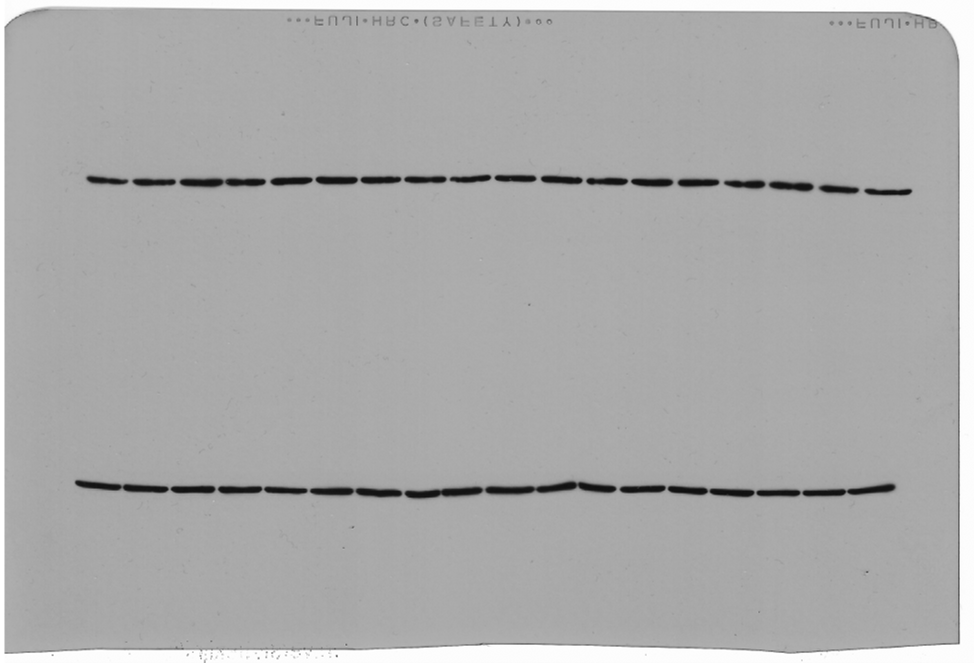

Supplement: Figure 5—source data 2. [file elife-96414-fig5-data2.zip › Figure 5 - source data 2/Fig5A_US27_Actin.tif]

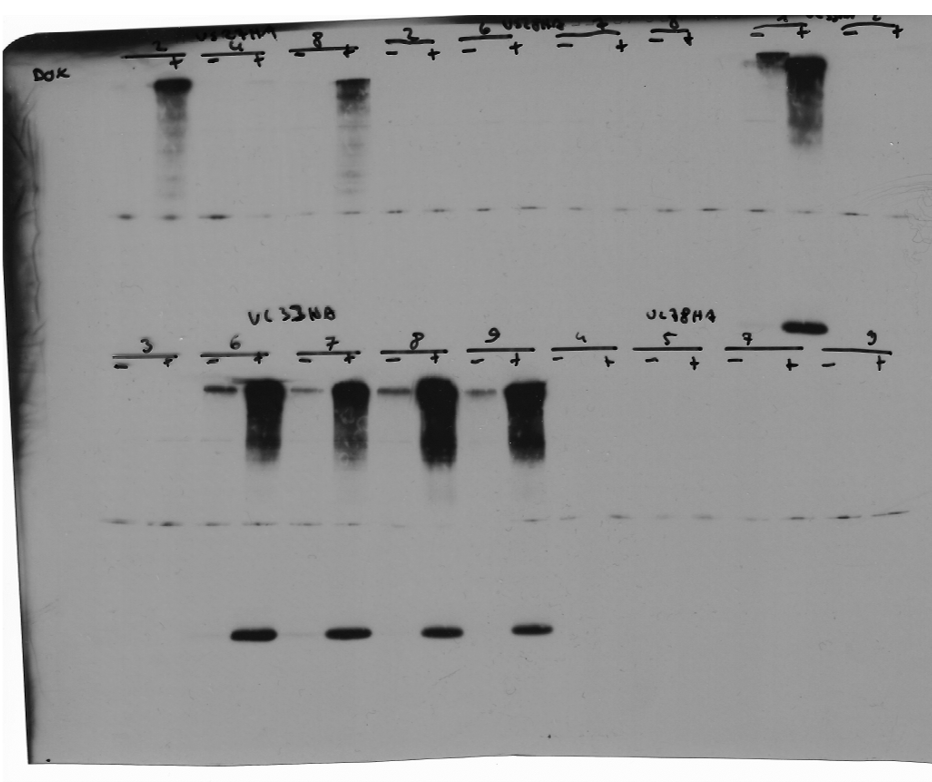

Supplement: Figure 5—source data 2. [file elife-96414-fig5-data2.zip › Figure 5 - source data 2/Fig5A_US27_HA.tif]

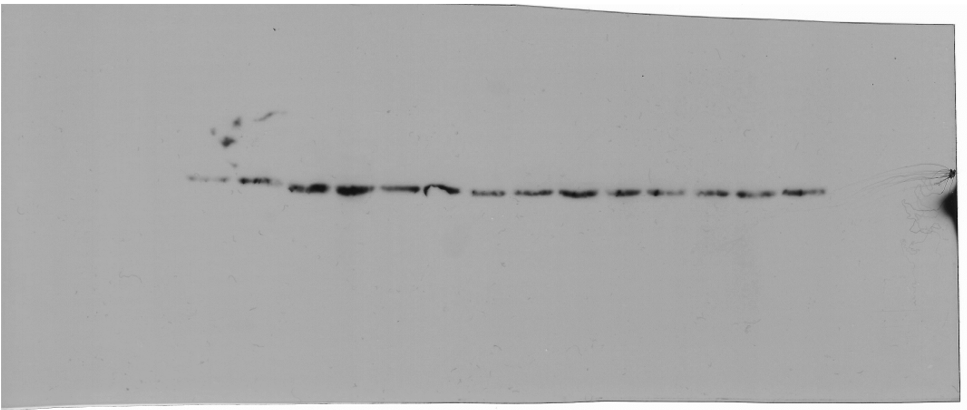

Supplement: Figure 5—source data 2. [file elife-96414-fig5-data2.zip › Figure 5 - source data 2/Fig5A_US28_Actin.tif]

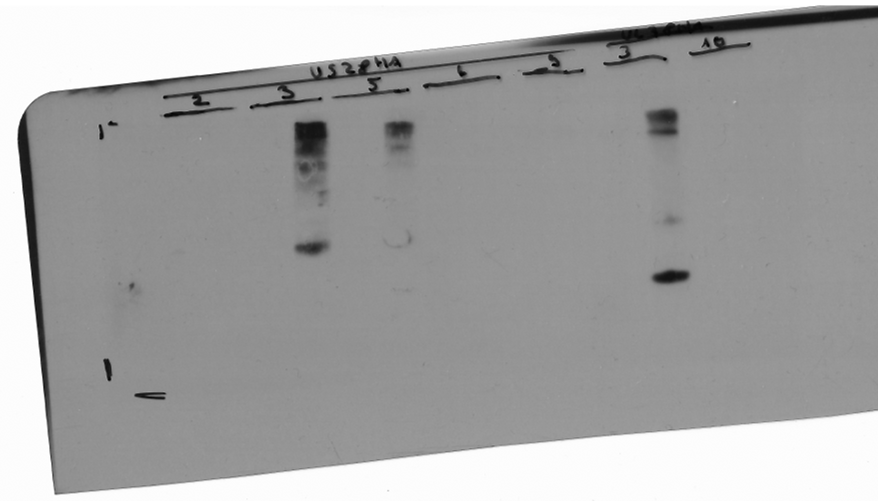

Supplement: Figure 5—source data 2. [file elife-96414-fig5-data2.zip › Figure 5 - source data 2/Fig5A_US28_HA.tif]

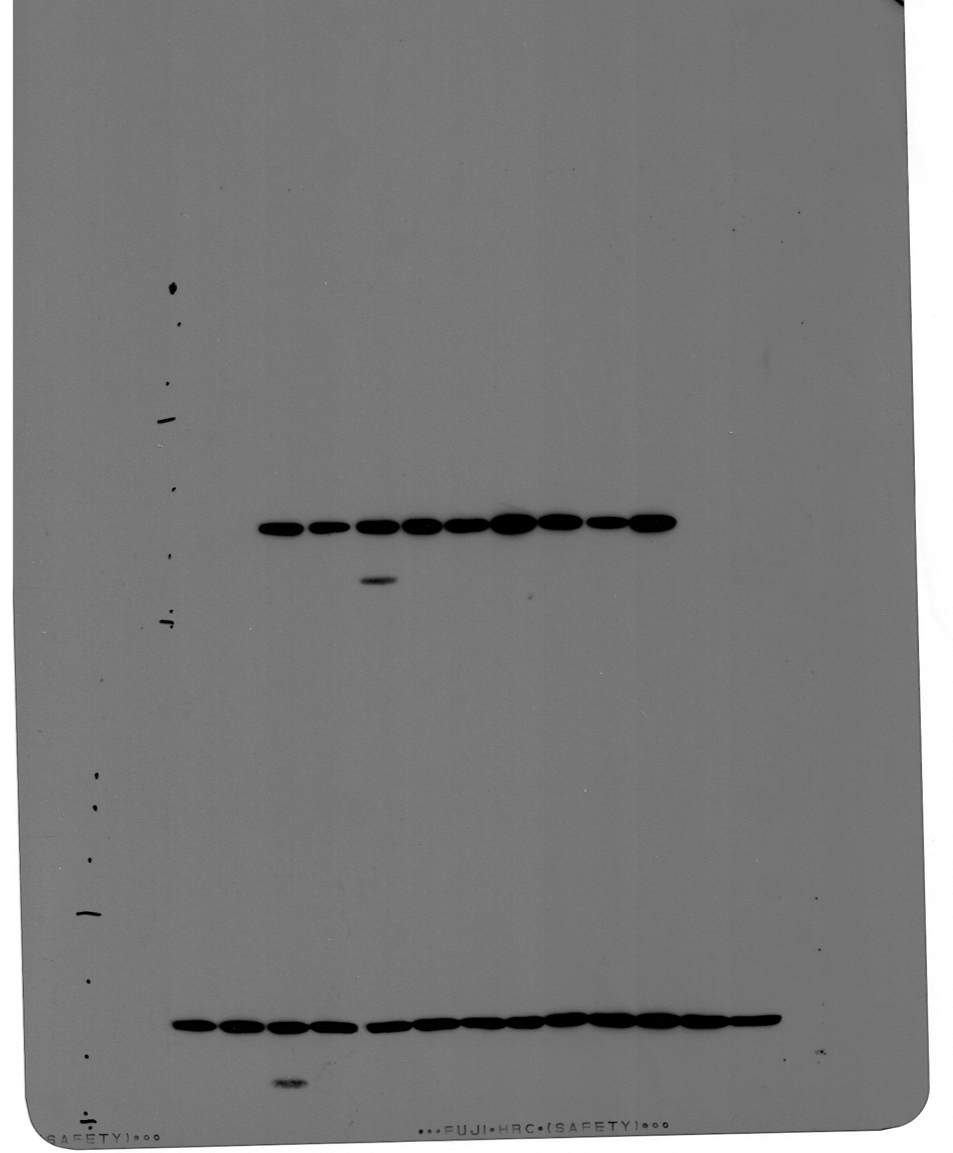

Supplement: Figure 5—source data 2. [file elife-96414-fig5-data2.zip › Figure 5 - source data 2/Fig5D_Actin.tif]

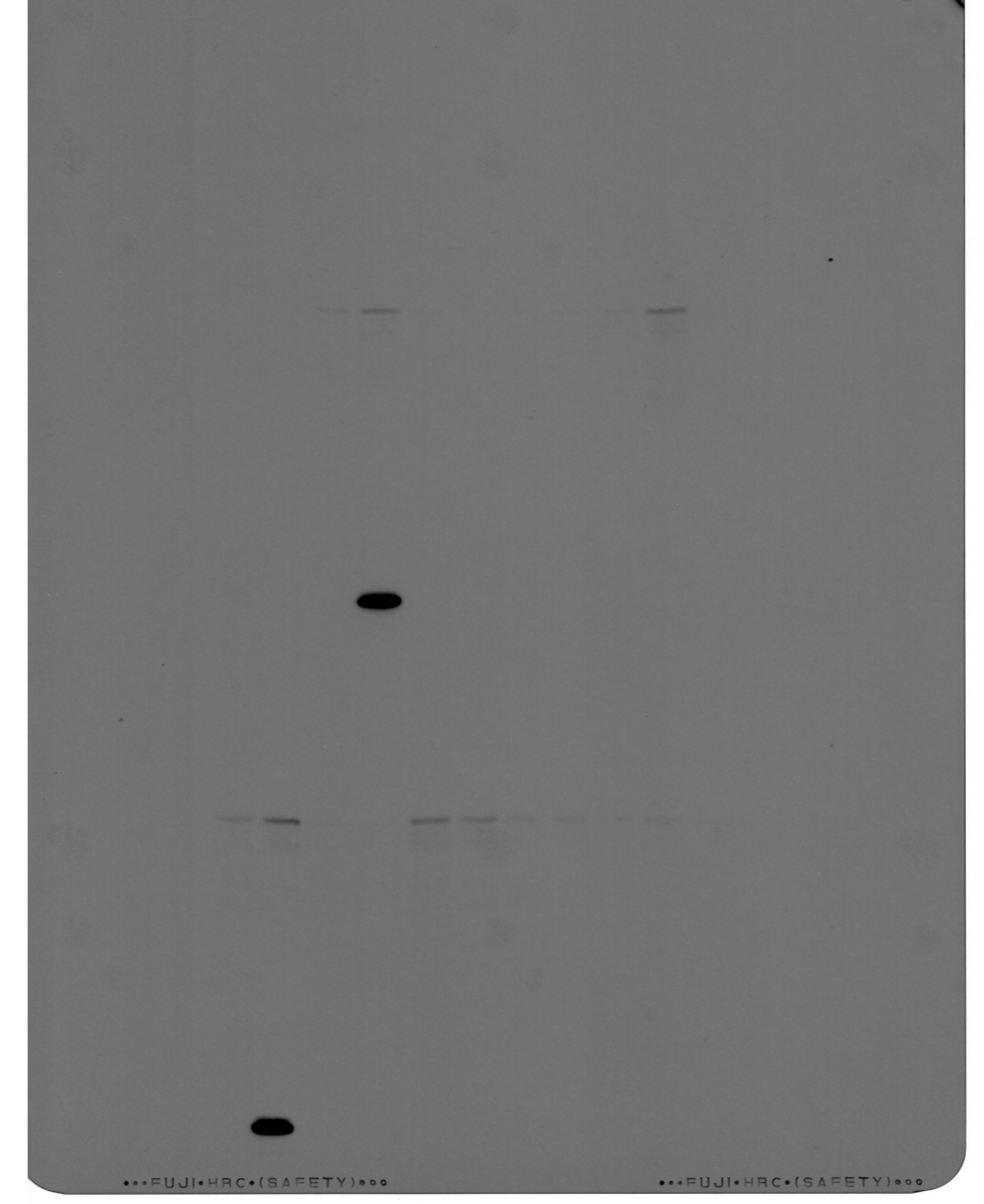

Supplement: Figure 5—source data 2. [file elife-96414-fig5-data2.zip › Figure 5 - source data 2/Fig5D_EYFP-Flag.tif]

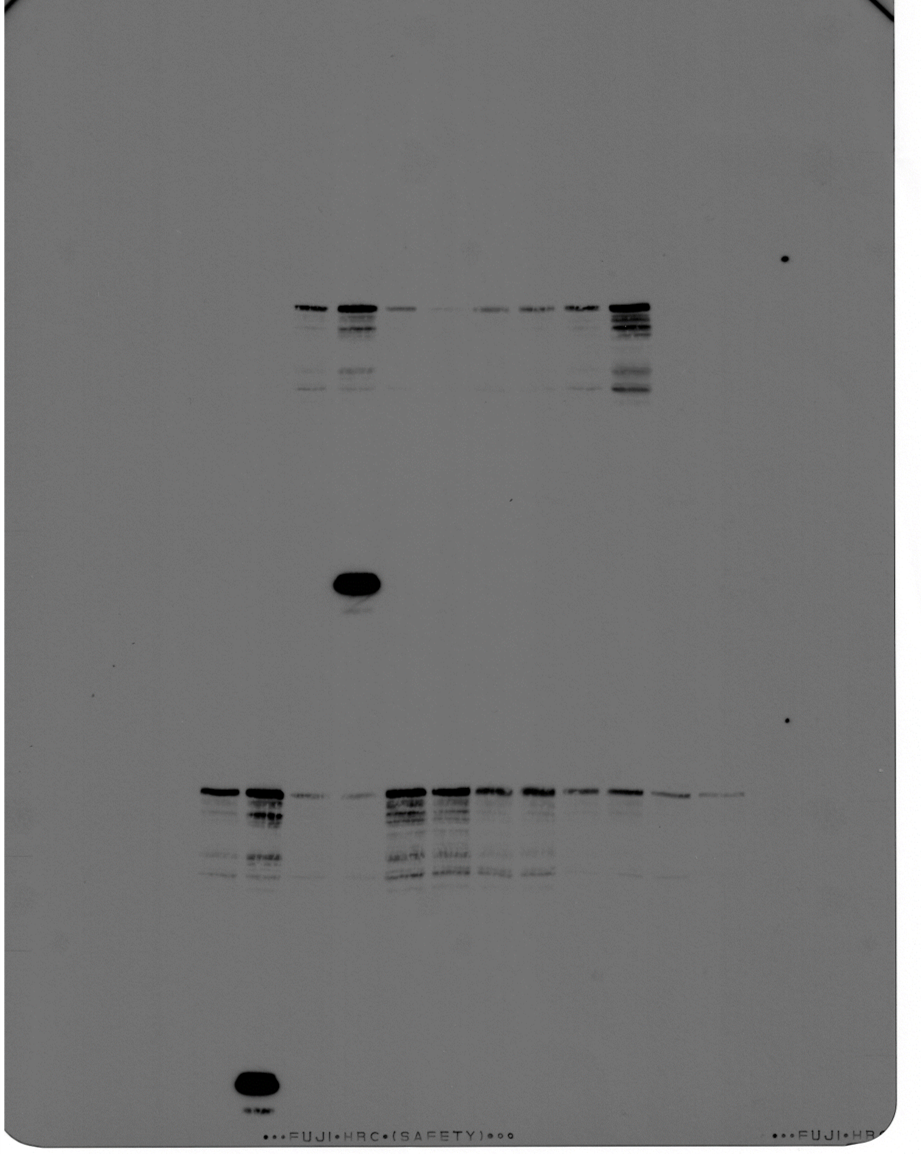

Supplement: Figure 5—source data 2. [file elife-96414-fig5-data2.zip › Figure 5 - source data 2/Fig5D_Flag.tif]

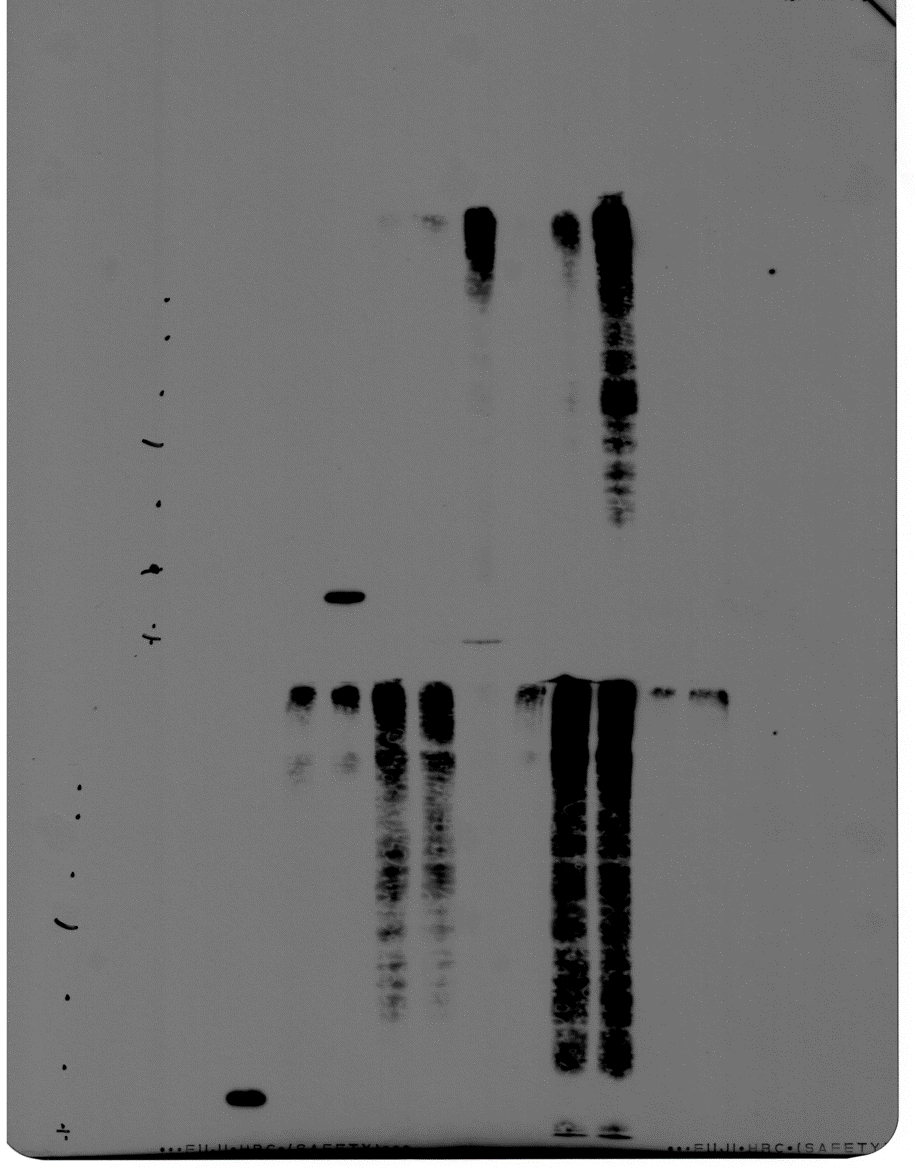

Supplement: Figure 5—source data 2. [file elife-96414-fig5-data2.zip › Figure 5 - source data 2/Fig5D_HA.tif]

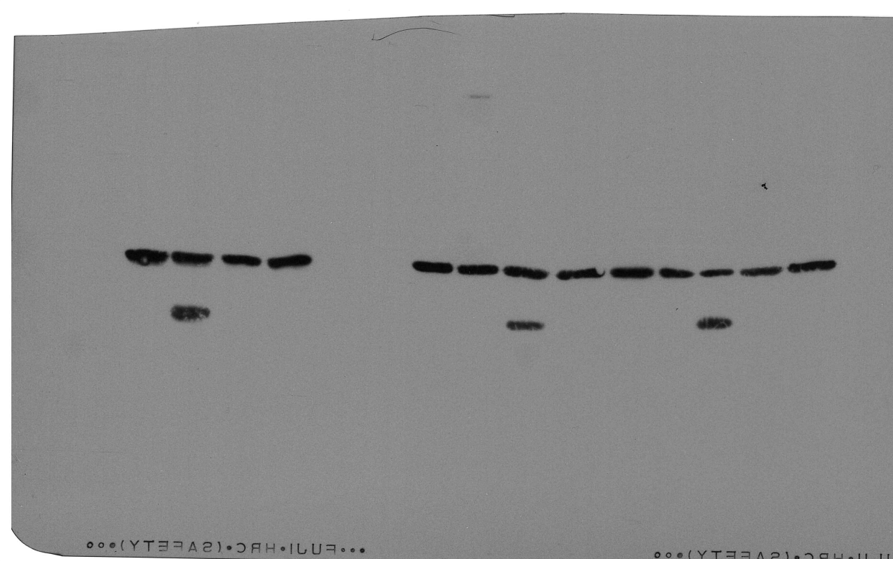

Supplement: Figure 5—source data 2. [file elife-96414-fig5-data2.zip › Figure 5 - source data 2/Fig5E_Actin.tif]

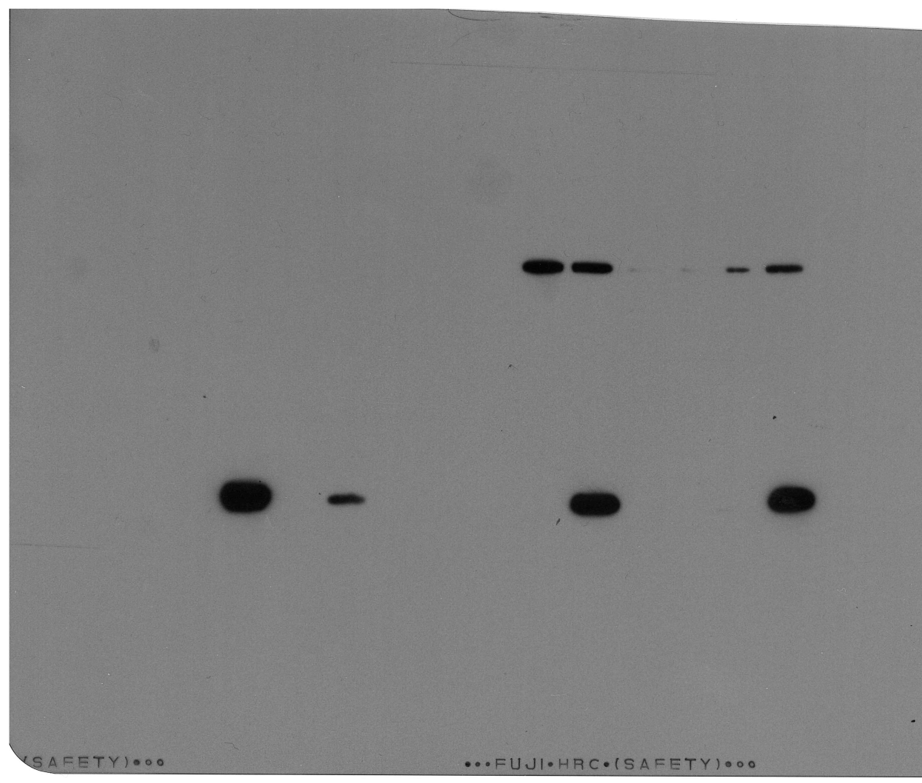

Supplement: Figure 5—source data 2. [file elife-96414-fig5-data2.zip › Figure 5 - source data 2/Fig5E_Flag.tif]

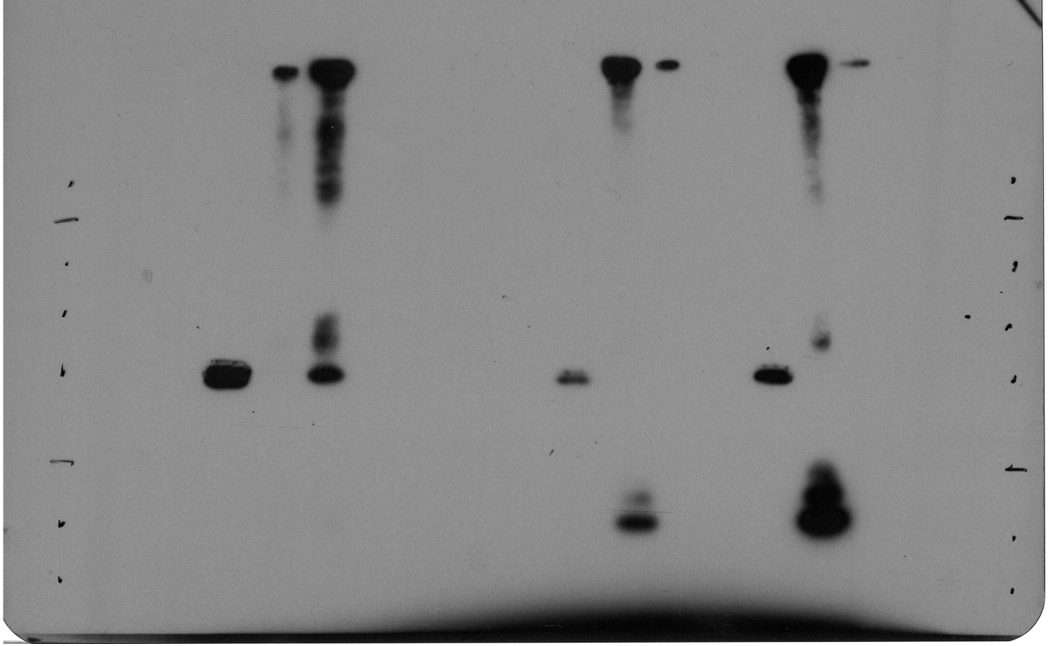

Supplement: Figure 5—source data 2. [file elife-96414-fig5-data2.zip › Figure 5 - source data 2/Fig5E_HA.tif]

Figure 6A

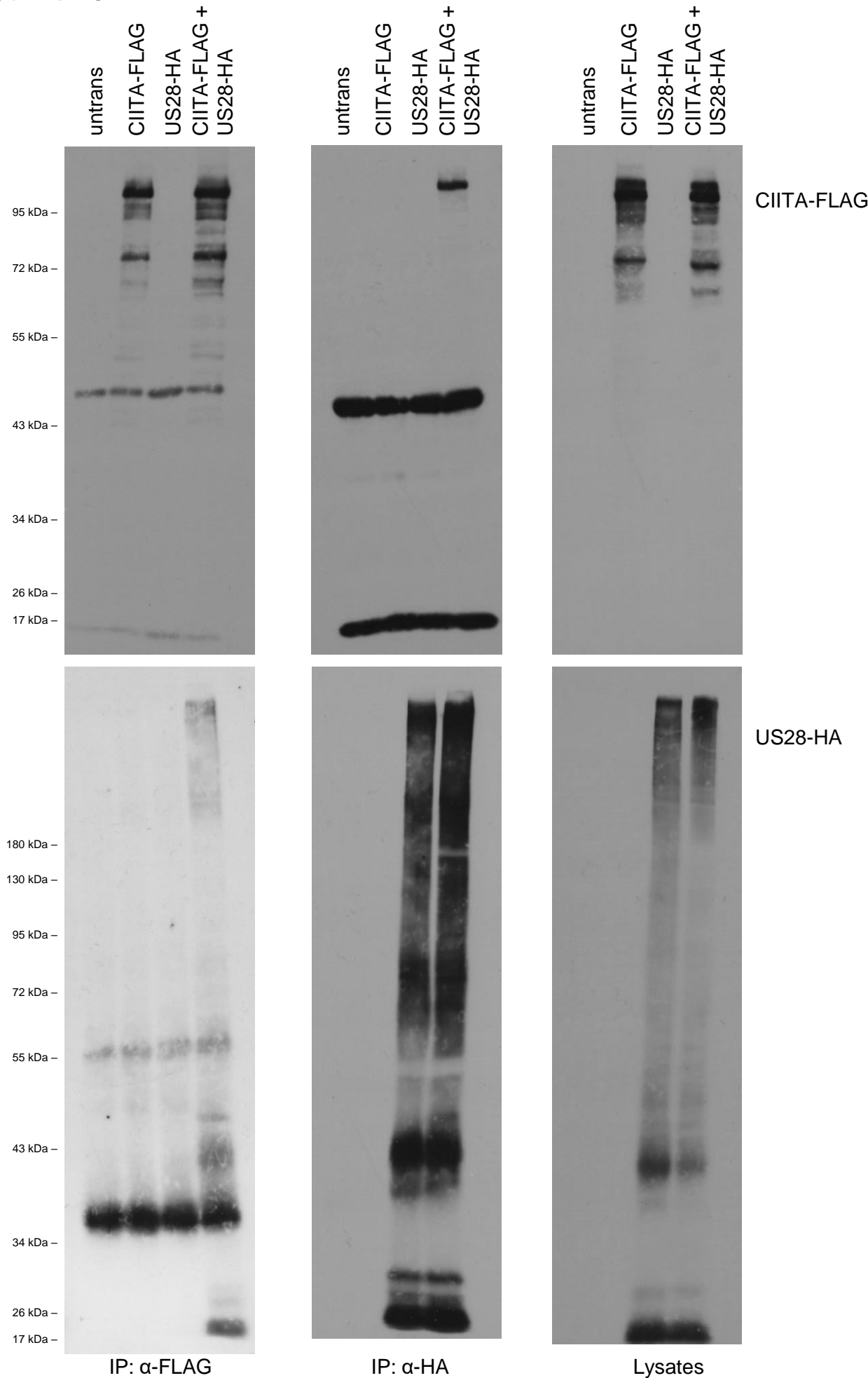

Supplement: Figure 6—source data 1. [file elife-96414-fig6-data1.zip › Figure 6 - source data 1/Fig.6A.pdf]

Figure 6B

Figure 6 – figure supplement 3

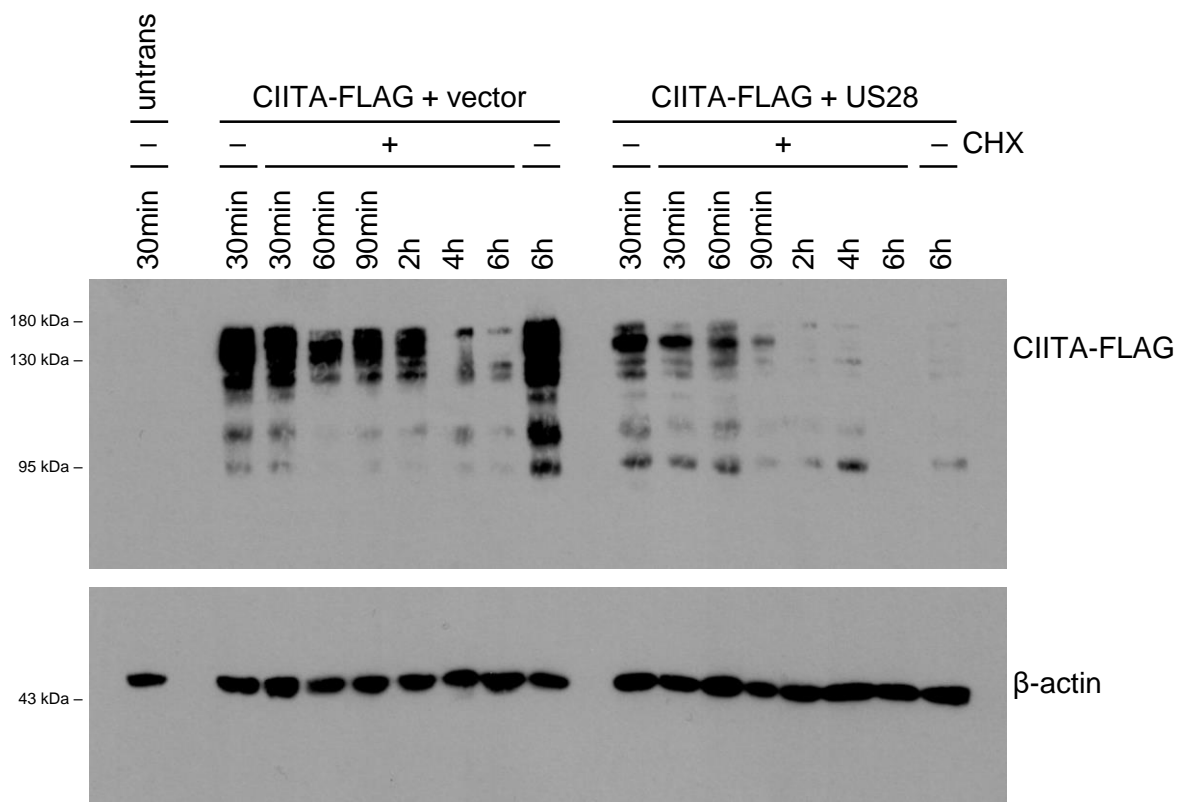

Supplement: Figure 6—source data 1. [file elife-96414-fig6-data1.zip › Figure 6 - source data 1/Fig.6B.pdf]

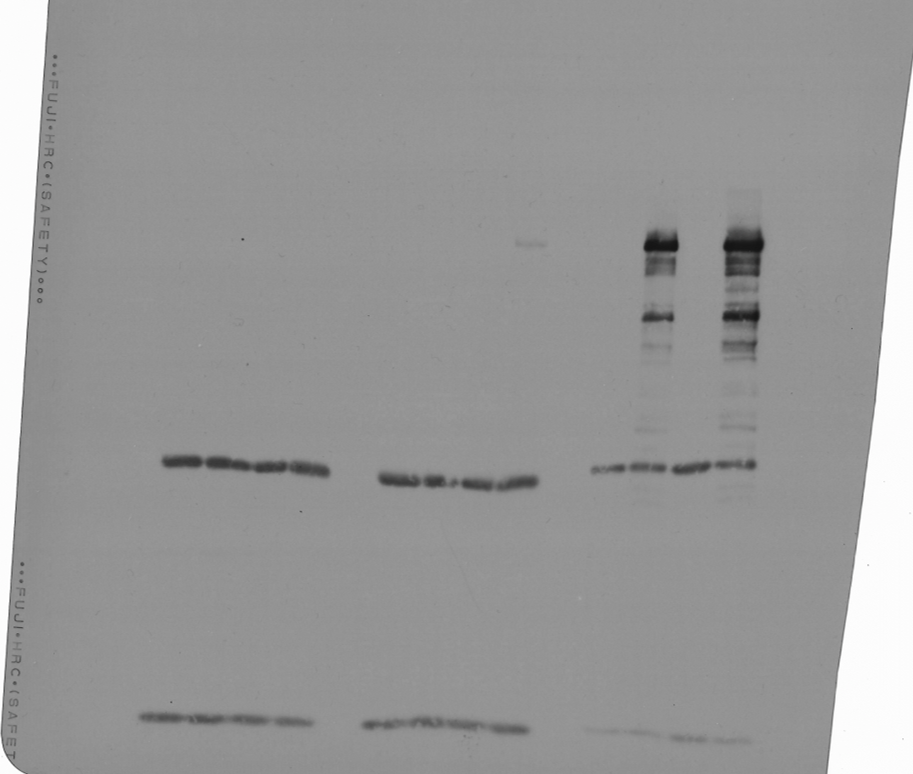

Supplement: Figure 6—source data 2. [file elife-96414-fig6-data2.zip › Figure 6 - source data 2/Fig6A_IP-Flag_Flag.tif]

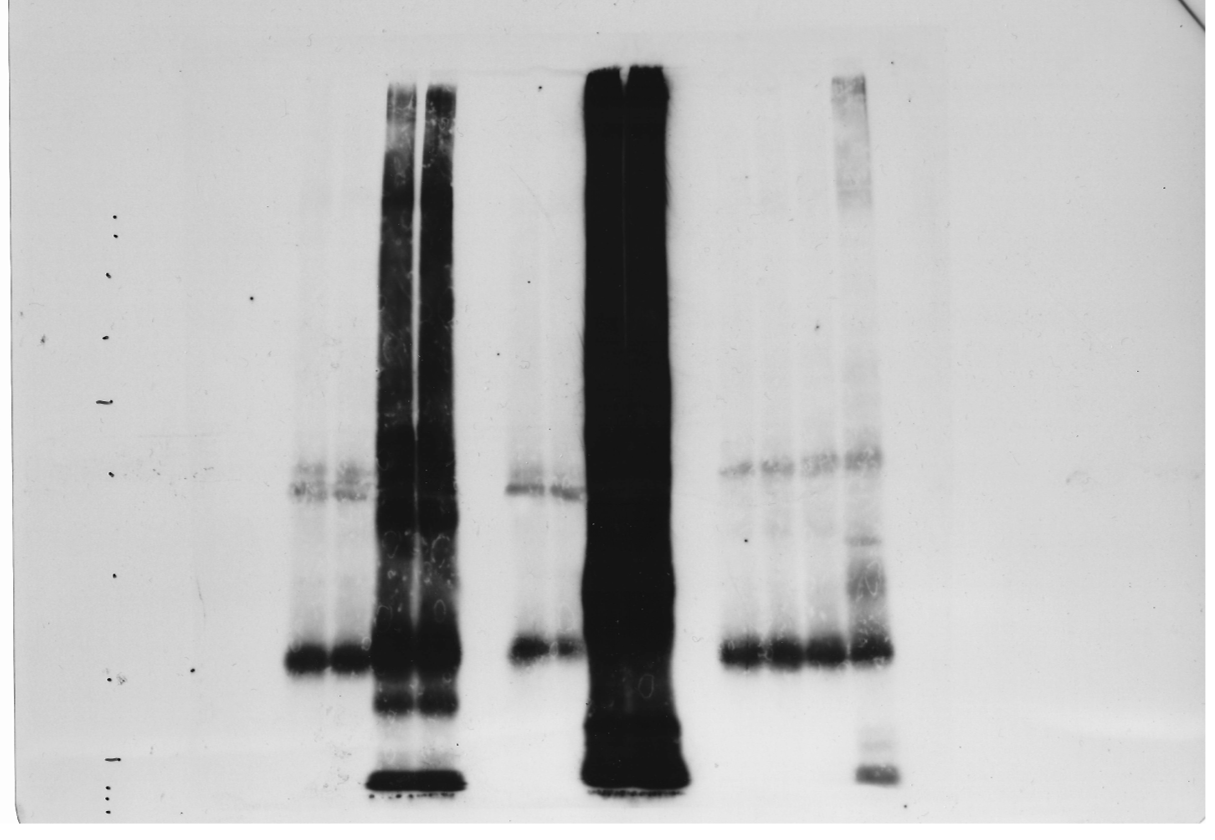

Supplement: Figure 6—source data 2. [file elife-96414-fig6-data2.zip › Figure 6 - source data 2/Fig6A_IP-Flag_HA.tif]

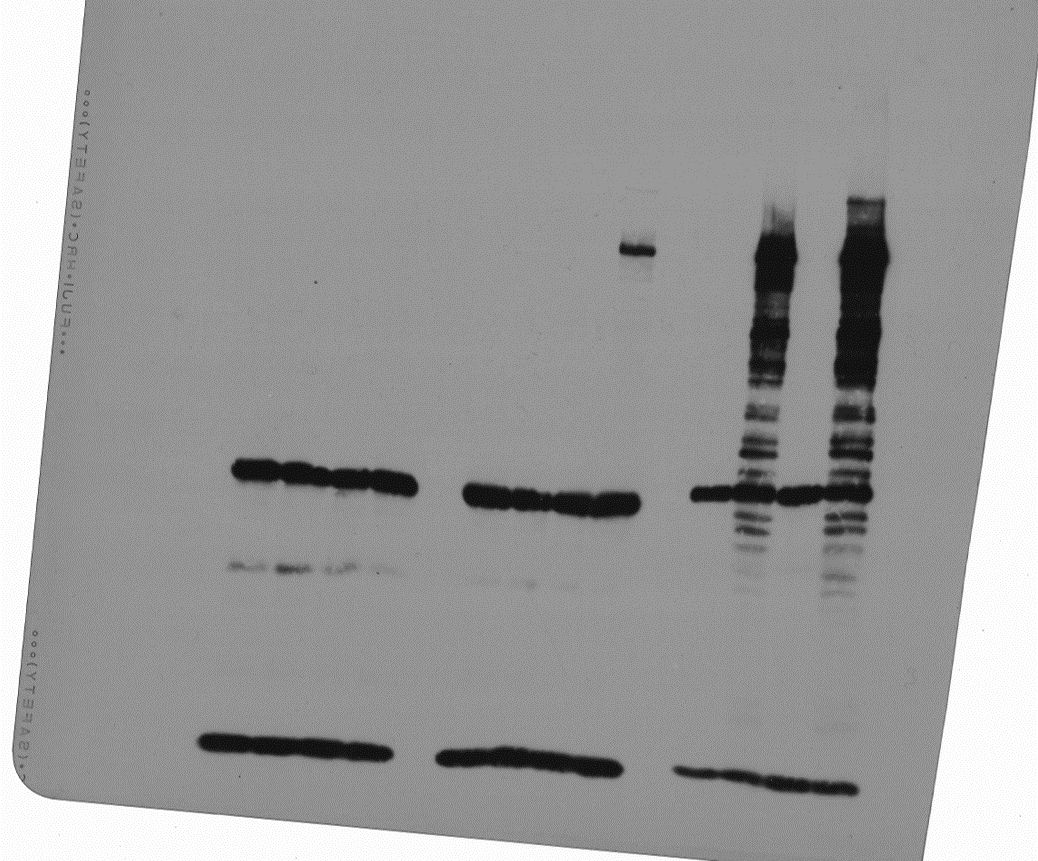

Supplement: Figure 6—source data 2. [file elife-96414-fig6-data2.zip › Figure 6 - source data 2/Fig6A_IP-HA_Flag.tif]

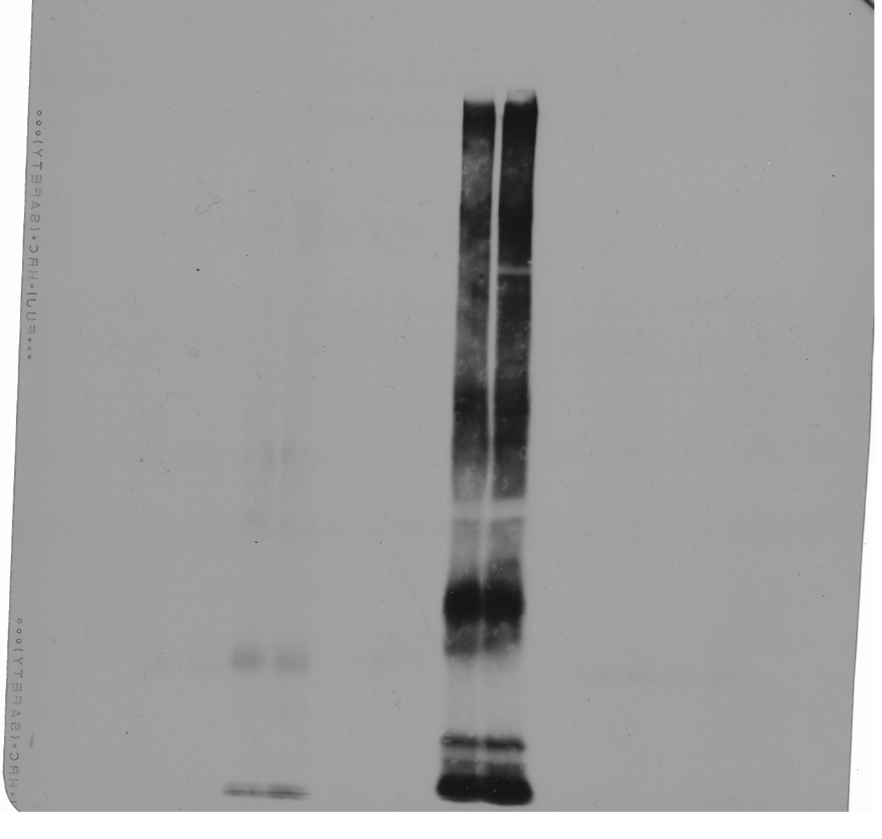

Supplement: Figure 6—source data 2. [file elife-96414-fig6-data2.zip › Figure 6 - source data 2/Fig6A_IP-HA_HA.tif]

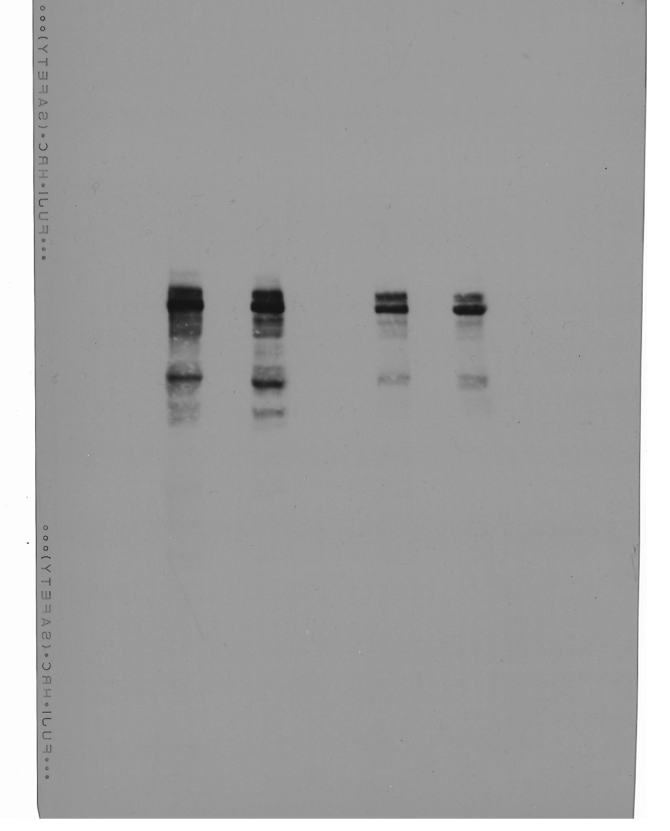

Supplement: Figure 6—source data 2. [file elife-96414-fig6-data2.zip › Figure 6 - source data 2/Fig6A_lysates_Flag.tif]

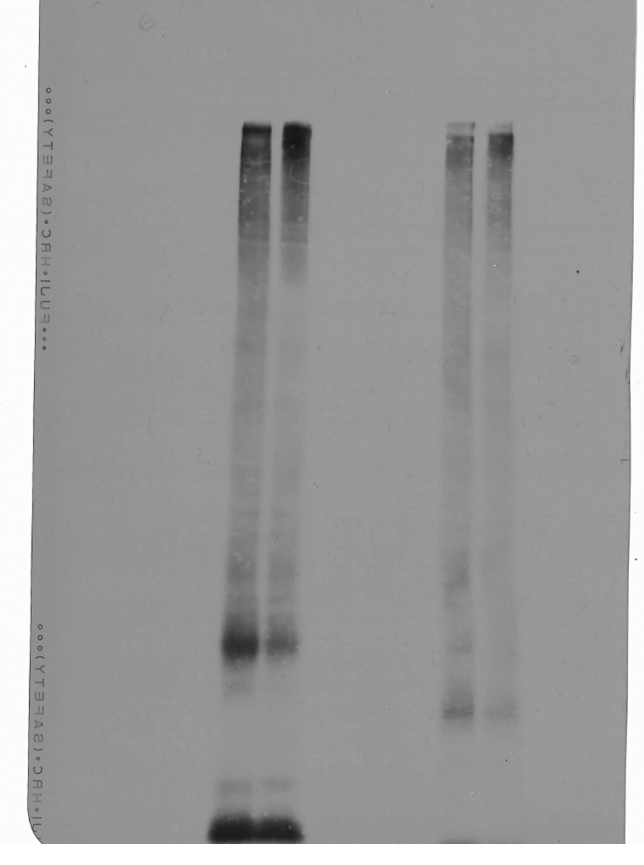

Supplement: Figure 6—source data 2. [file elife-96414-fig6-data2.zip › Figure 6 - source data 2/Fig6A_lysates_HA.tif]

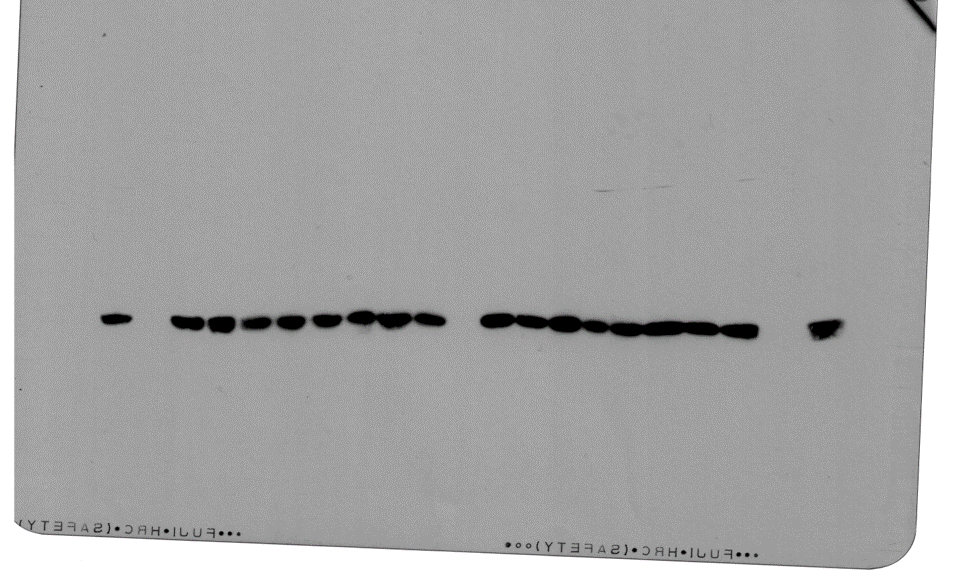

Supplement: Figure 6—source data 2. [file elife-96414-fig6-data2.zip › Figure 6 - source data 2/Fig6B_Actin.tif]

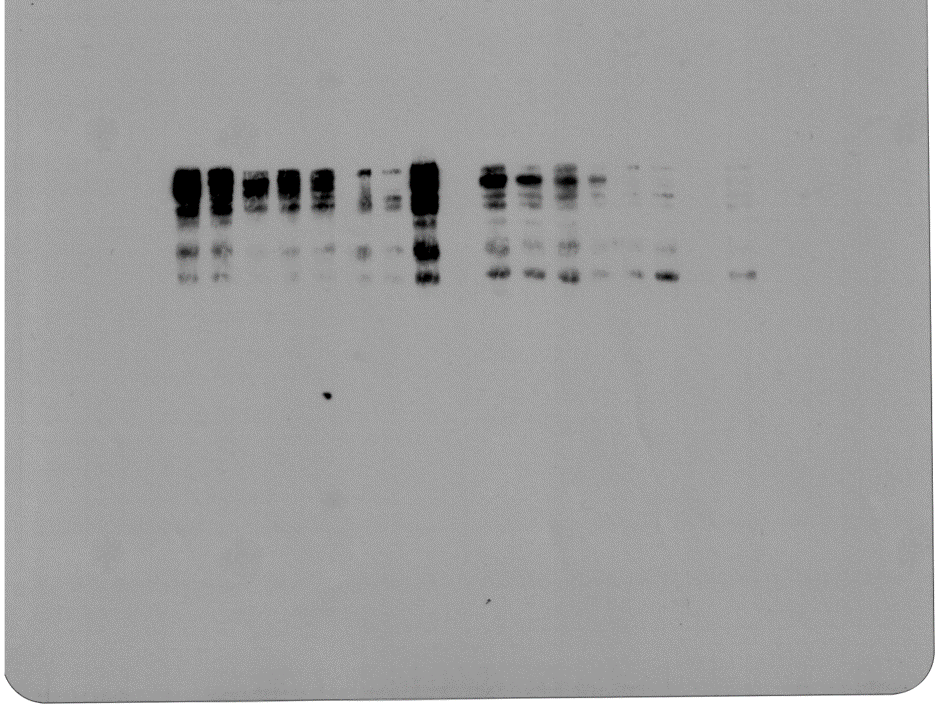

Supplement: Figure 6—source data 2. [file elife-96414-fig6-data2.zip › Figure 6 - source data 2/Fig6B_Flag.tif]

Figure 6 – figure supplement 1

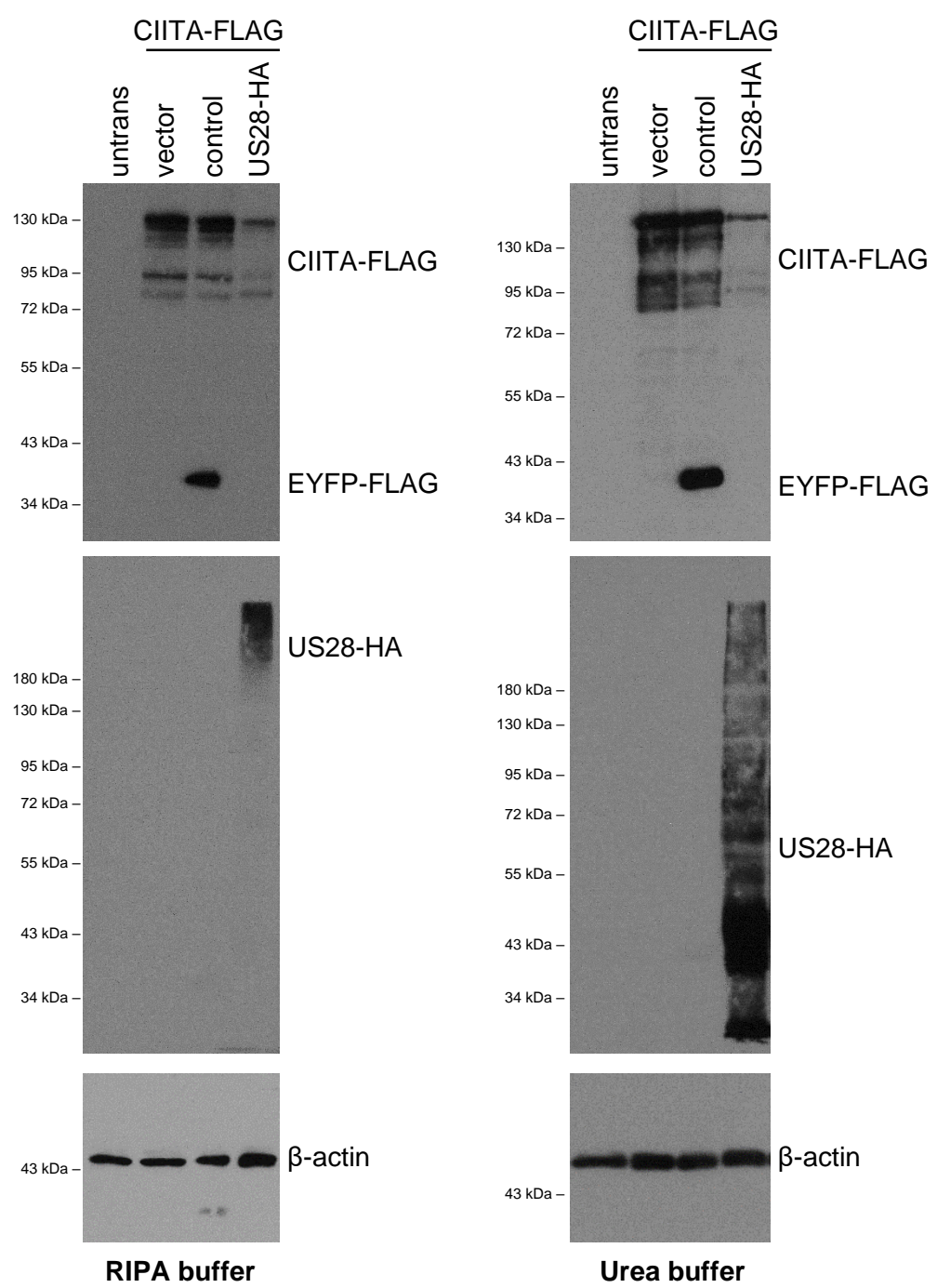

Supplement: Figure 6—figure supplement 1—source data 1. [file elife-96414-fig6-figsupp1-data1.zip › Figure 6 - figure supplement 1 - source data 1/Fig.6_fig.suppl.1.pdf]

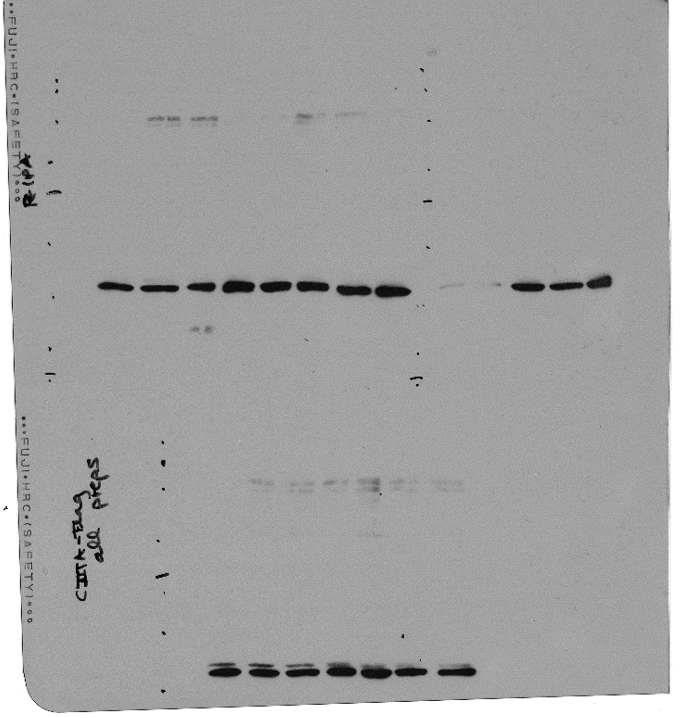

Supplement: Figure 6—figure supplement 1—source data 2. [file elife-96414-fig6-figsupp1-data2.zip › Figure 6 - figure supplement 1 - source data 2/Fig6_Supp1_RIPA_Actin.tif]

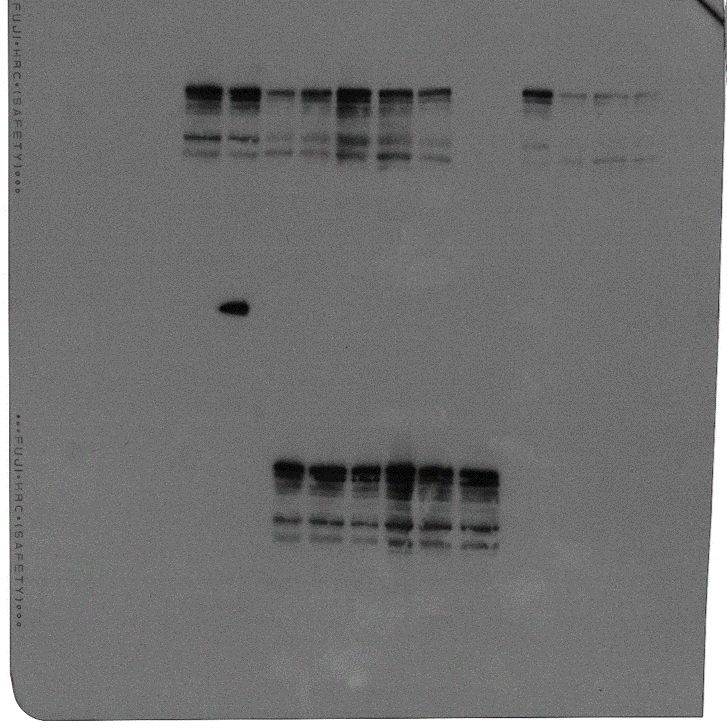

Supplement: Figure 6—figure supplement 1—source data 2. [file elife-96414-fig6-figsupp1-data2.zip › Figure 6 - figure supplement 1 - source data 2/Fig6_Supp1_RIPA_Flag.tif]

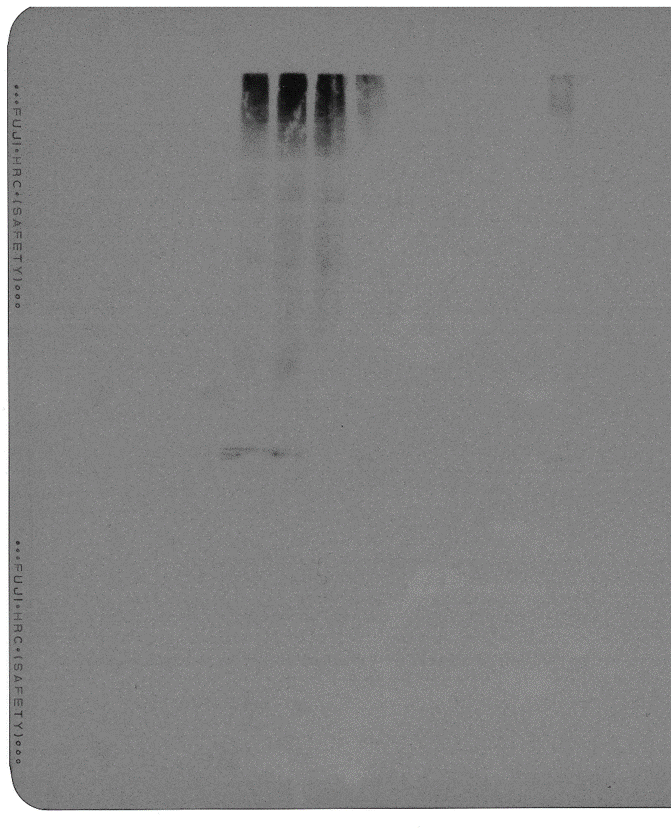

Supplement: Figure 6—figure supplement 1—source data 2. [file elife-96414-fig6-figsupp1-data2.zip › Figure 6 - figure supplement 1 - source data 2/Fig6_Supp1_RIPA_HA.tif]

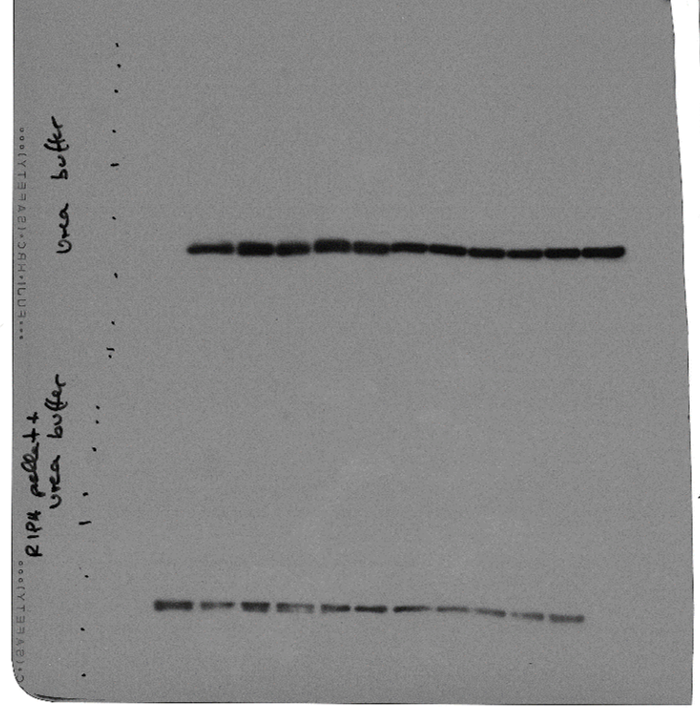

Supplement: Figure 6—figure supplement 1—source data 2. [file elife-96414-fig6-figsupp1-data2.zip › Figure 6 - figure supplement 1 - source data 2/Fig6_Supp1_Urea_Actin.tif]

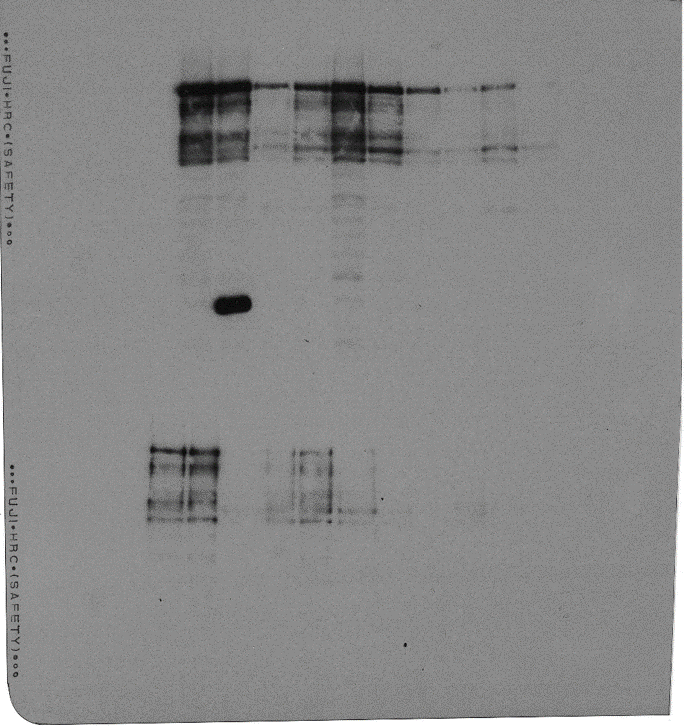

Supplement: Figure 6—figure supplement 1—source data 2. [file elife-96414-fig6-figsupp1-data2.zip › Figure 6 - figure supplement 1 - source data 2/Fig6_Supp1_Urea_Flag.tif]

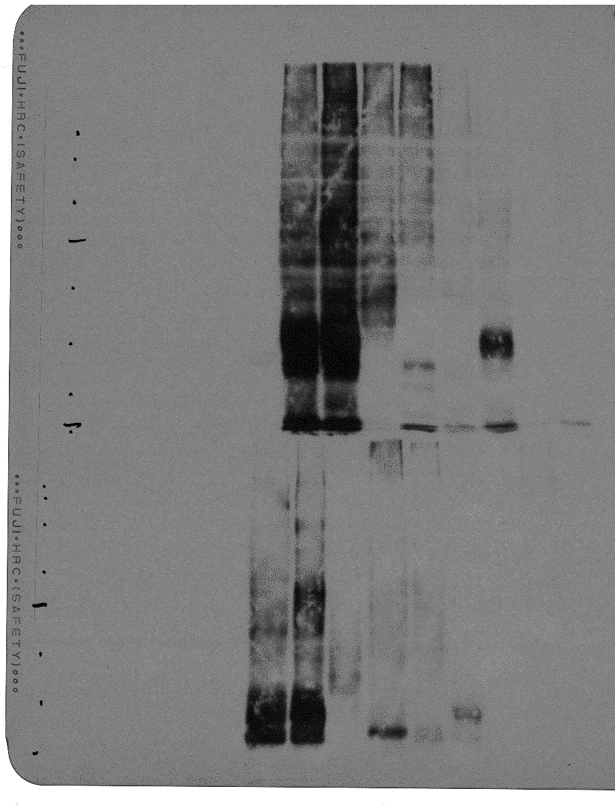

Supplement: Figure 6—figure supplement 1—source data 2. [file elife-96414-fig6-figsupp1-data2.zip › Figure 6 - figure supplement 1 - source data 2/Fig6_Supp1_Urea_HA.tif]

Figure 6 – figure supplement 2

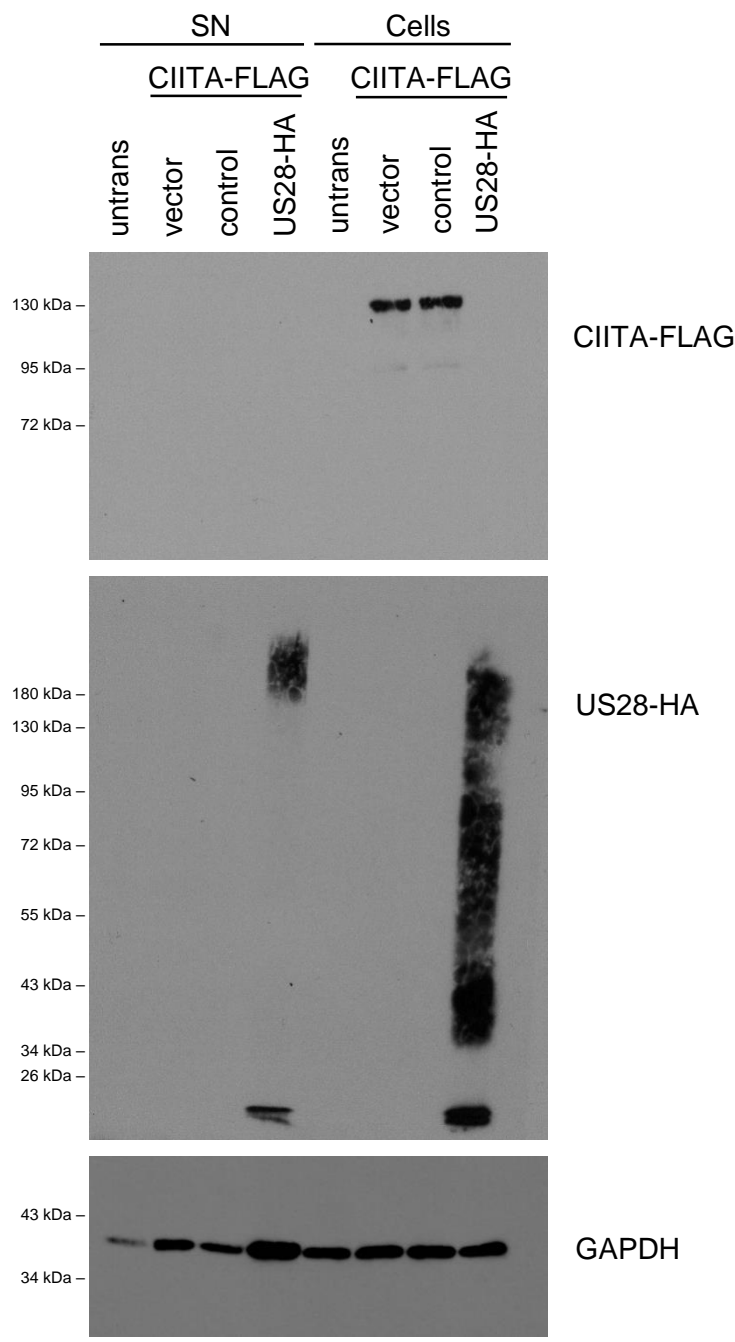

Supplement: Figure 6—figure supplement 2—source data 1. [file elife-96414-fig6-figsupp2-data1.zip › Figure 6 - figure supplement 2 - source data 1/Fig.6_fig.suppl.2.pdf]

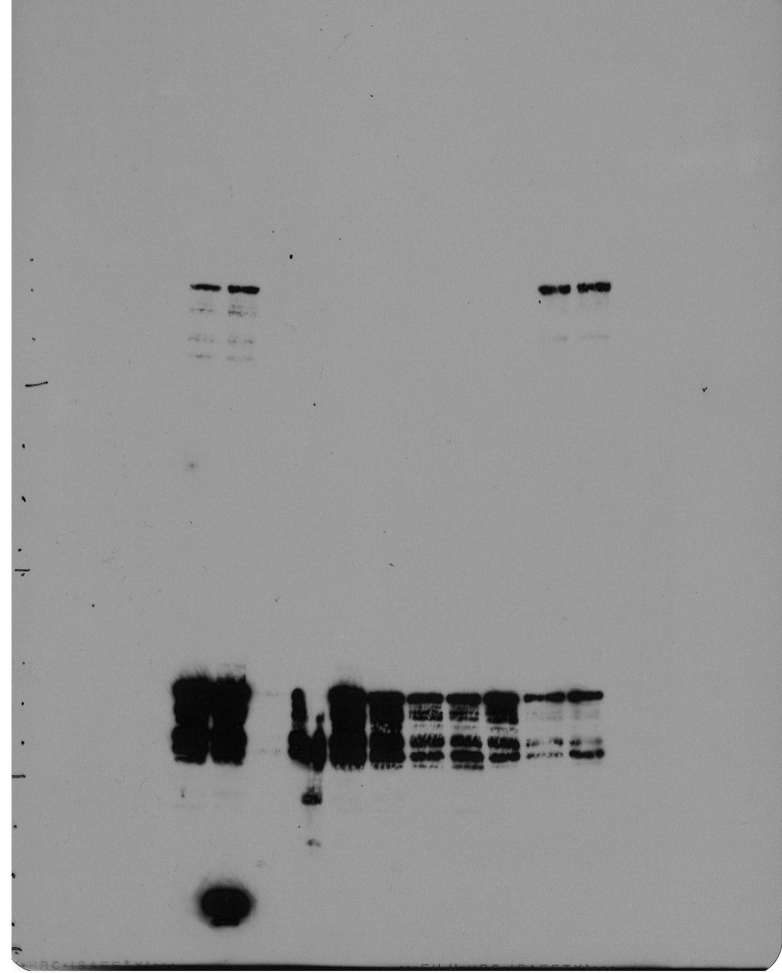

Supplement: Figure 6—figure supplement 2—source data 2. [file elife-96414-fig6-figsupp2-data2.zip › Figure 6 - figure supplement 2 - source data 2/Fig6_Supp2_Flag.tif]

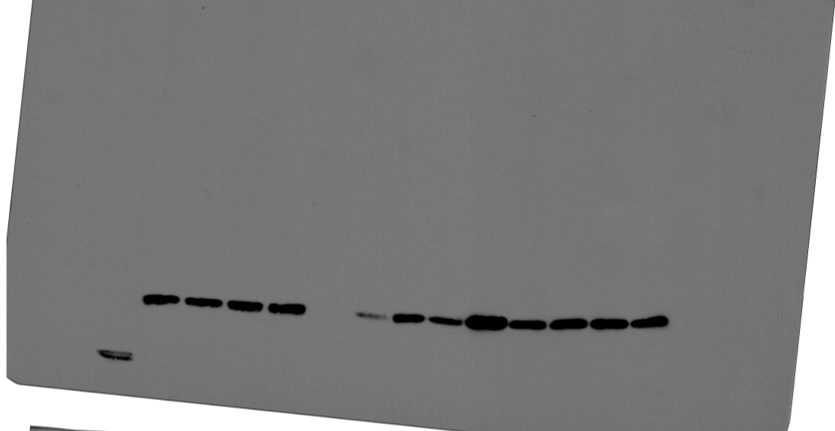

Supplement: Figure 6—figure supplement 2—source data 2. [file elife-96414-fig6-figsupp2-data2.zip › Figure 6 - figure supplement 2 - source data 2/Fig6_Supp2_GAPDH.tif]

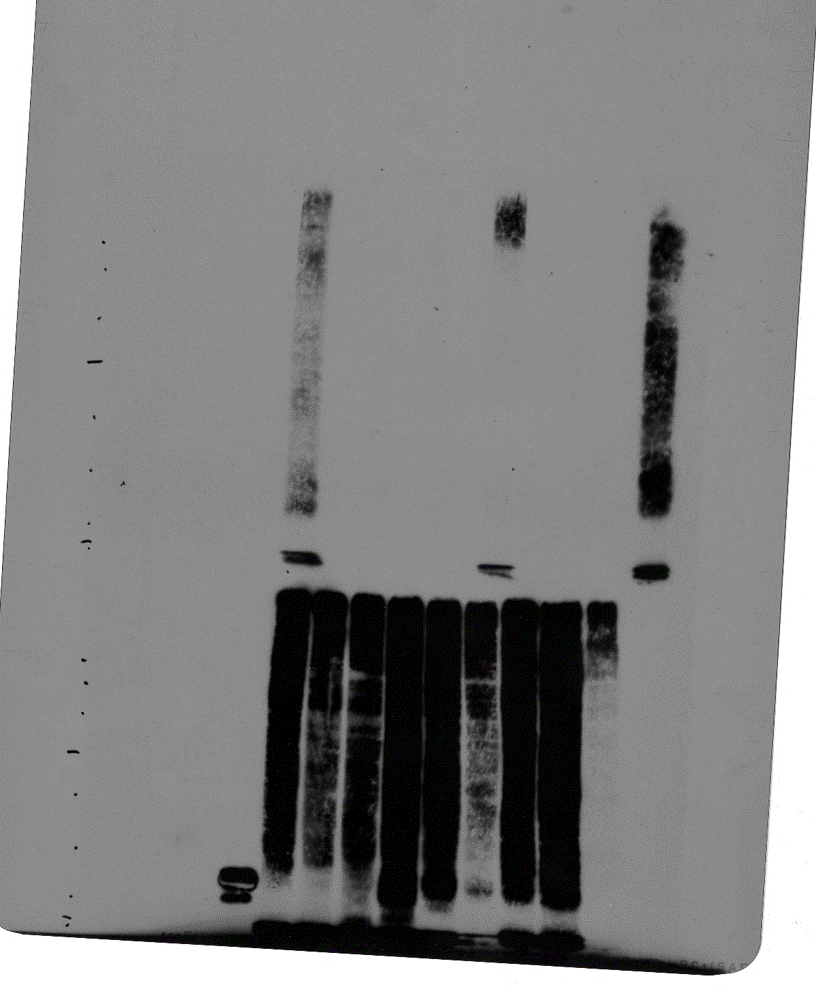

Supplement: Figure 6—figure supplement 2—source data 2. [file elife-96414-fig6-figsupp2-data2.zip › Figure 6 - figure supplement 2 - source data 2/Fig6_Supp2_HA.tif]

Figure 6 – figure supplement 4 A

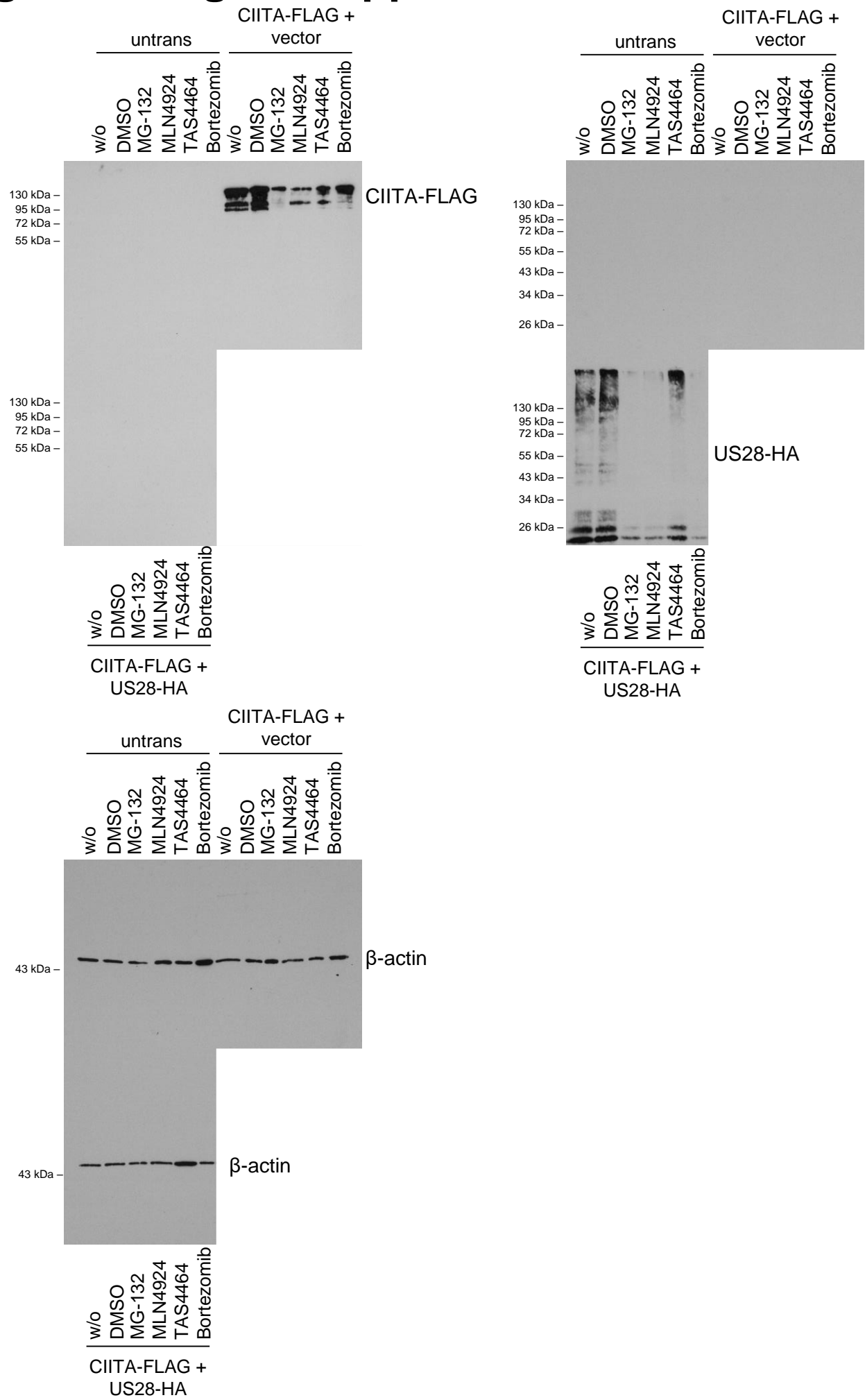

Supplement: Figure 6—figure supplement 4—source data 1. [file elife-96414-fig6-figsupp4-data1.zip › Figure 6 - figure supplement 4 - source data 1/Fig.6_fig.suppl.4A.pdf]

Figure 6 – figure supplement 4 B

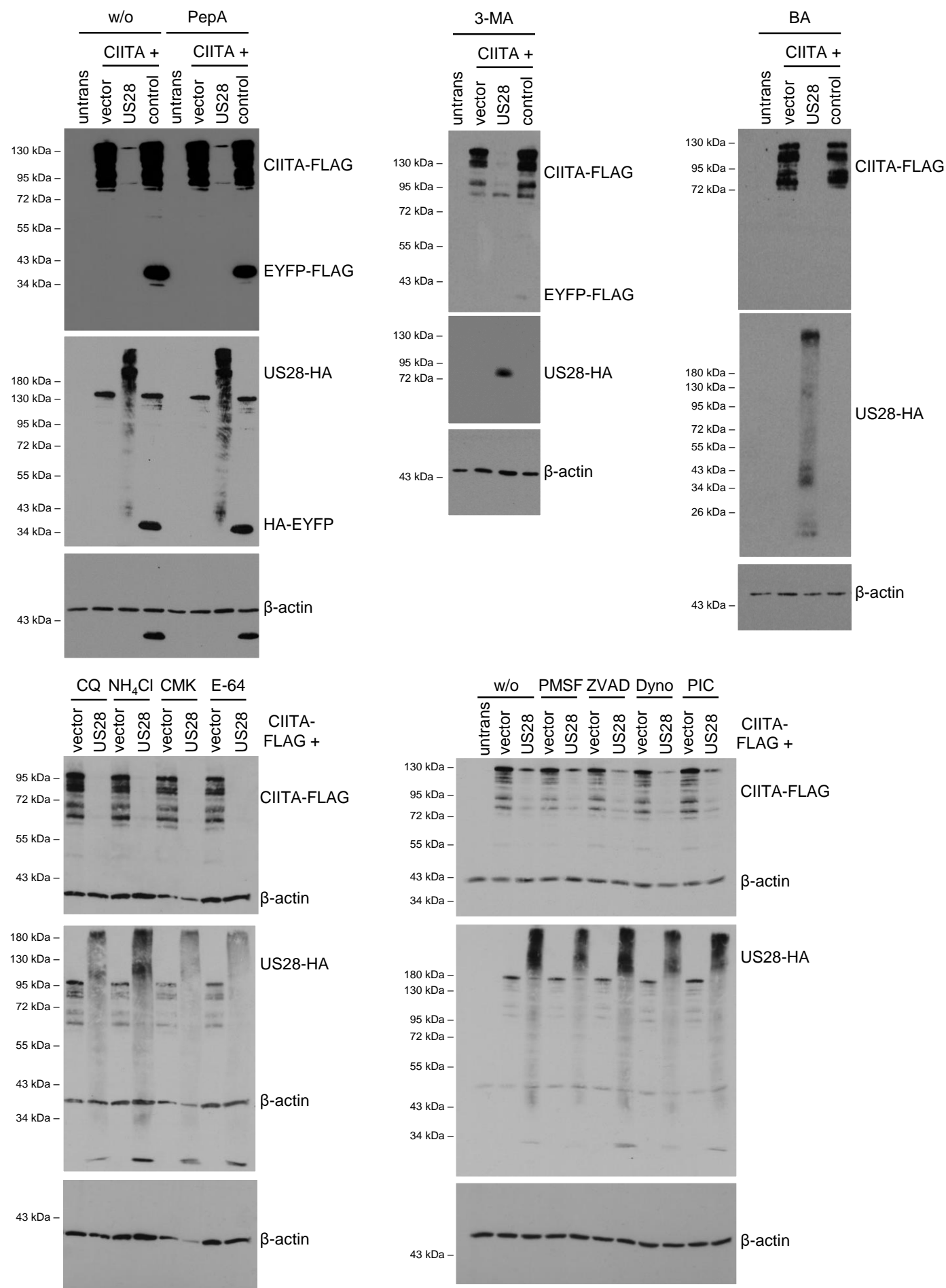

Supplement: Figure 6—figure supplement 4—source data 1. [file elife-96414-fig6-figsupp4-data1.zip › Figure 6 - figure supplement 4 - source data 1/Fig.6_fig.suppl.4B.pdf]

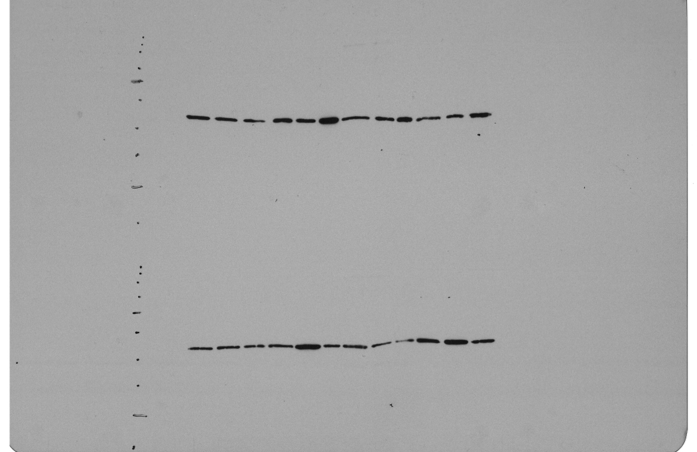

Supplement: Figure 6—figure supplement 4—source data 2. [file elife-96414-fig6-figsupp4-data2.zip › Figure 6 - figure supplement 4 - source data 2/Fig6_Supp4A_Actin.tif]

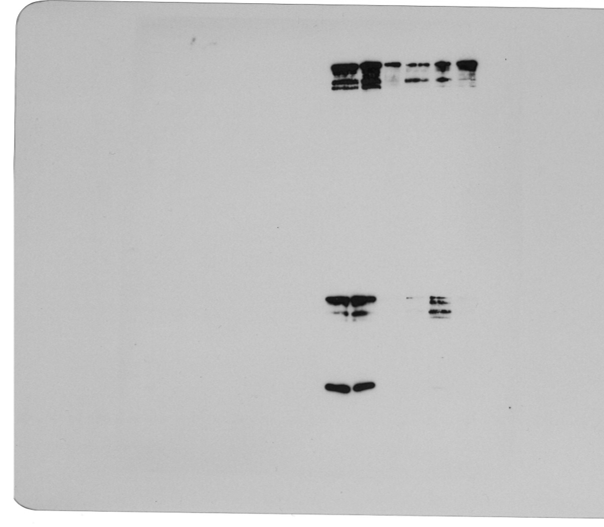

Supplement: Figure 6—figure supplement 4—source data 2. [file elife-96414-fig6-figsupp4-data2.zip › Figure 6 - figure supplement 4 - source data 2/Fig6_Supp4A_Flag.tif]

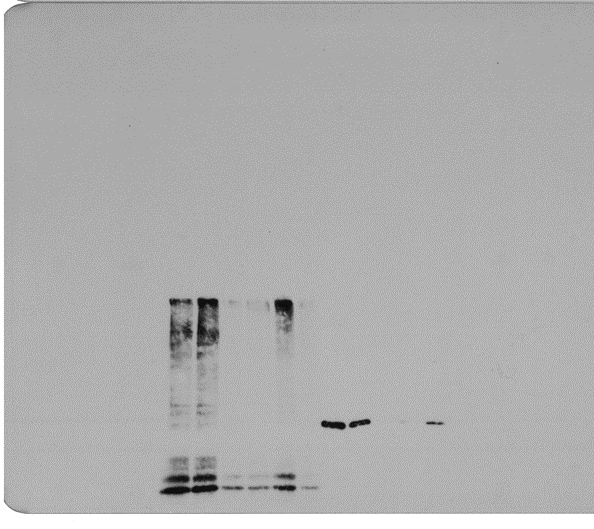

Supplement: Figure 6—figure supplement 4—source data 2. [file elife-96414-fig6-figsupp4-data2.zip › Figure 6 - figure supplement 4 - source data 2/Fig6_Supp4A_HA.tif]

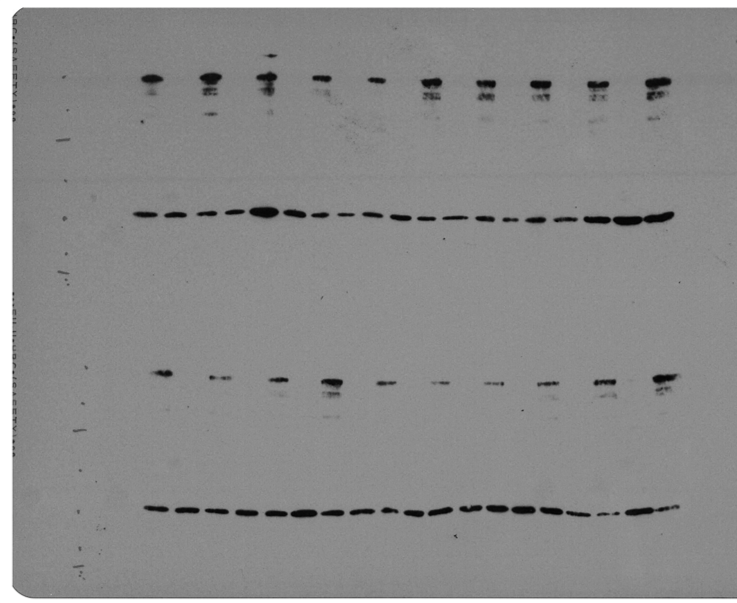

Supplement: Figure 6—figure supplement 4—source data 2. [file elife-96414-fig6-figsupp4-data2.zip › Figure 6 - figure supplement 4 - source data 2/Fig6_Supp4B_3MA_Actin.tif]

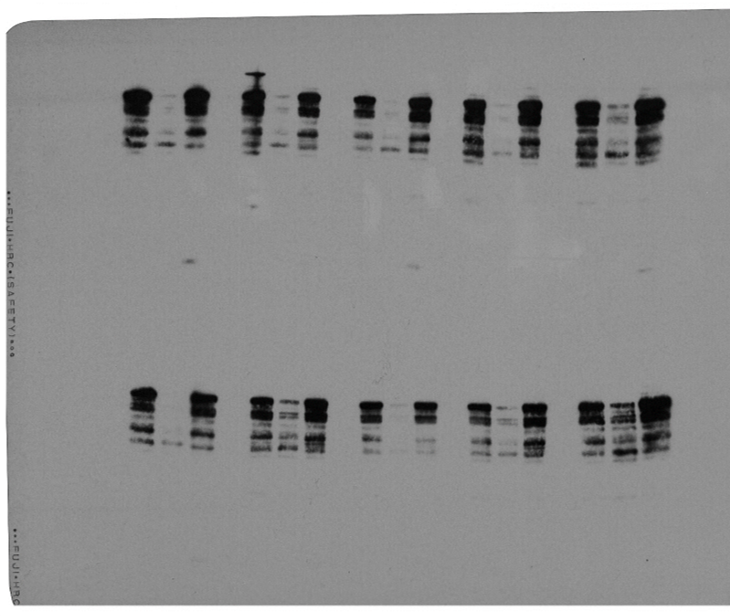

Supplement: Figure 6—figure supplement 4—source data 2. [file elife-96414-fig6-figsupp4-data2.zip › Figure 6 - figure supplement 4 - source data 2/Fig6_Supp4B_3MA_Flag.tif]

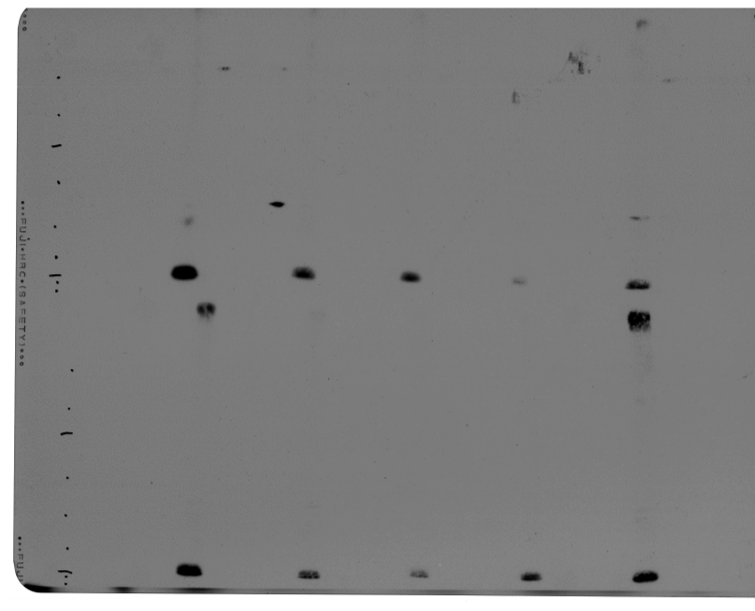

Supplement: Figure 6—figure supplement 4—source data 2. [file elife-96414-fig6-figsupp4-data2.zip › Figure 6 - figure supplement 4 - source data 2/Fig6_Supp4B_3MA_HA.tif]

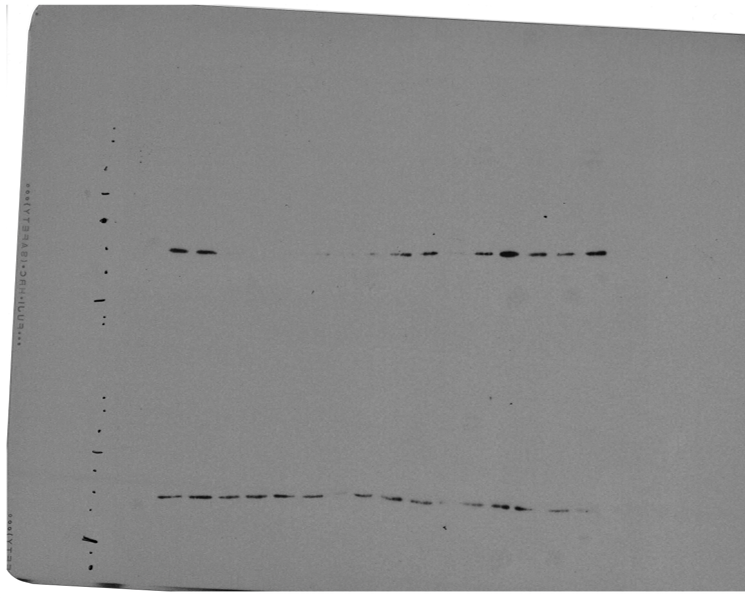

Supplement: Figure 6—figure supplement 4—source data 2. [file elife-96414-fig6-figsupp4-data2.zip › Figure 6 - figure supplement 4 - source data 2/Fig6_Supp4B_BA_Actin.tif]

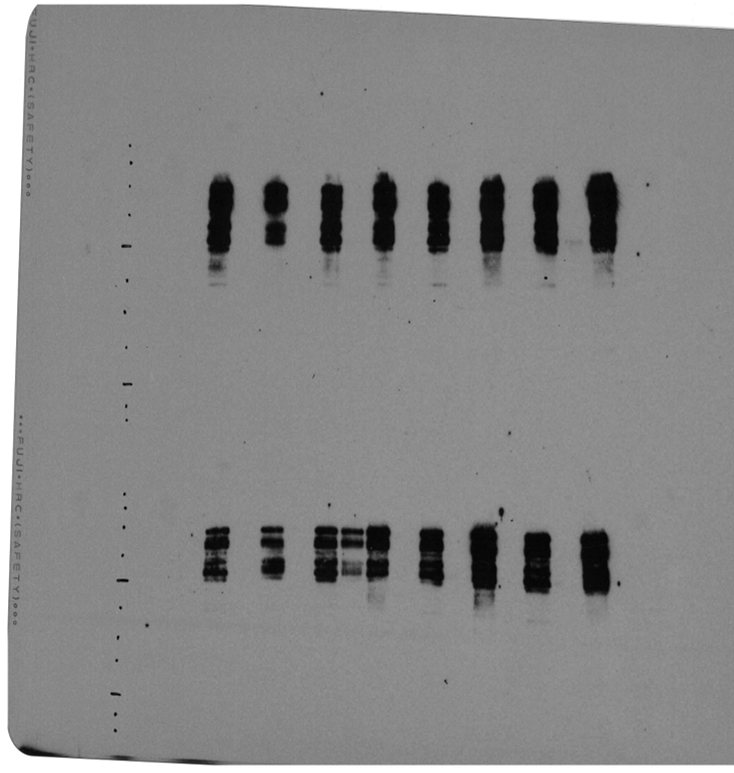

Supplement: Figure 6—figure supplement 4—source data 2. [file elife-96414-fig6-figsupp4-data2.zip › Figure 6 - figure supplement 4 - source data 2/Fig6_Supp4B_BA_Flag.tif]

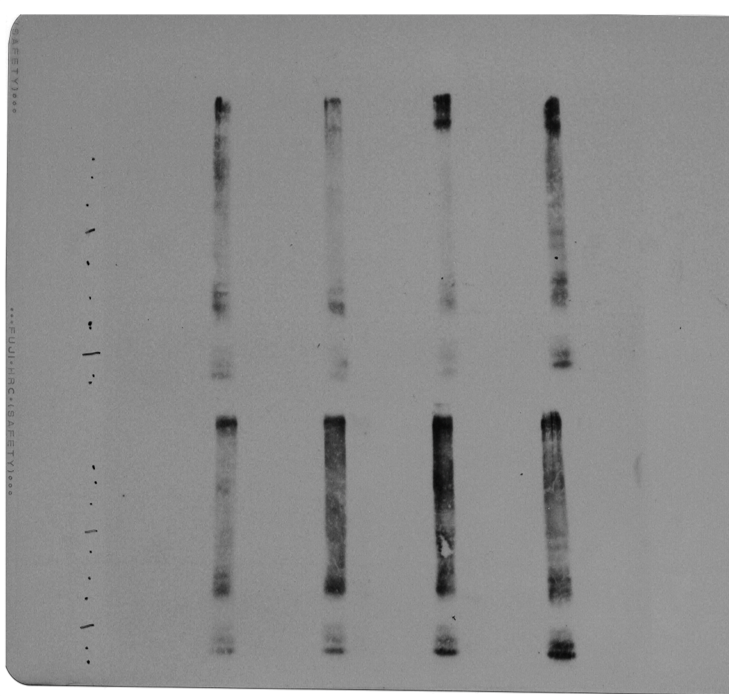

Supplement: Figure 6—figure supplement 4—source data 2. [file elife-96414-fig6-figsupp4-data2.zip › Figure 6 - figure supplement 4 - source data 2/Fig6_Supp4B_BA_HA.tif]

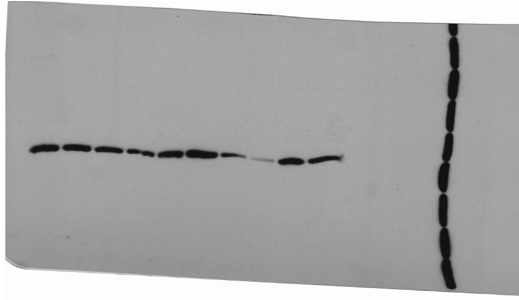

Supplement: Figure 6—figure supplement 4—source data 2. [file elife-96414-fig6-figsupp4-data2.zip › Figure 6 - figure supplement 4 - source data 2/Fig6_Supp4B_CQ-NH4Cl-CMK-E64_Actin.tif]

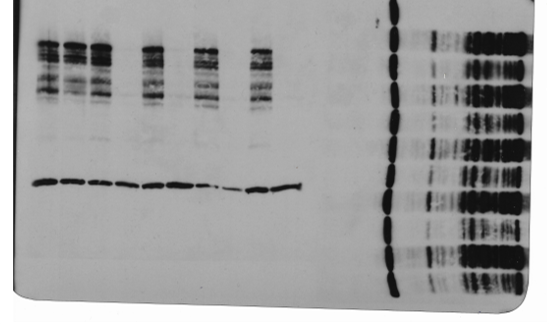

Supplement: Figure 6—figure supplement 4—source data 2. [file elife-96414-fig6-figsupp4-data2.zip › Figure 6 - figure supplement 4 - source data 2/Fig6_Supp4B_CQ-NH4Cl-CMK-E64_Flag.tif]

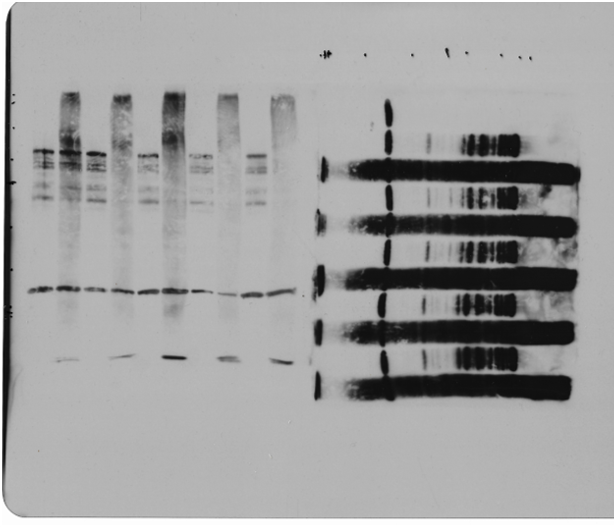

Supplement: Figure 6—figure supplement 4—source data 2. [file elife-96414-fig6-figsupp4-data2.zip › Figure 6 - figure supplement 4 - source data 2/Fig6_Supp4B_CQ-NH4Cl-CMK-E64_HA.tif]

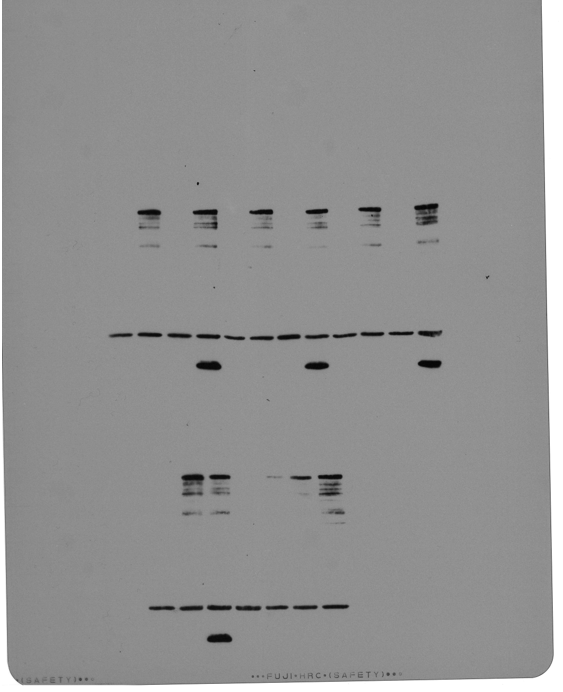

Supplement: Figure 6—figure supplement 4—source data 2. [file elife-96414-fig6-figsupp4-data2.zip › Figure 6 - figure supplement 4 - source data 2/Fig6_Supp4B_PepA_Actin.tif]

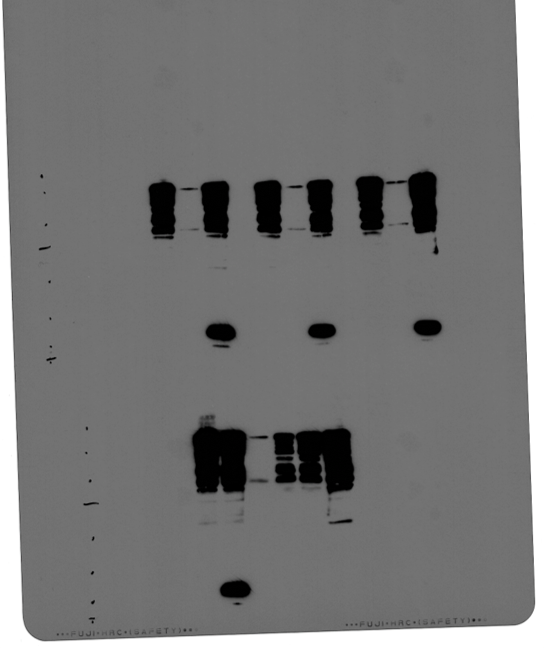

Supplement: Figure 6—figure supplement 4—source data 2. [file elife-96414-fig6-figsupp4-data2.zip › Figure 6 - figure supplement 4 - source data 2/Fig6_Supp4B_PepA_Flag.tif]

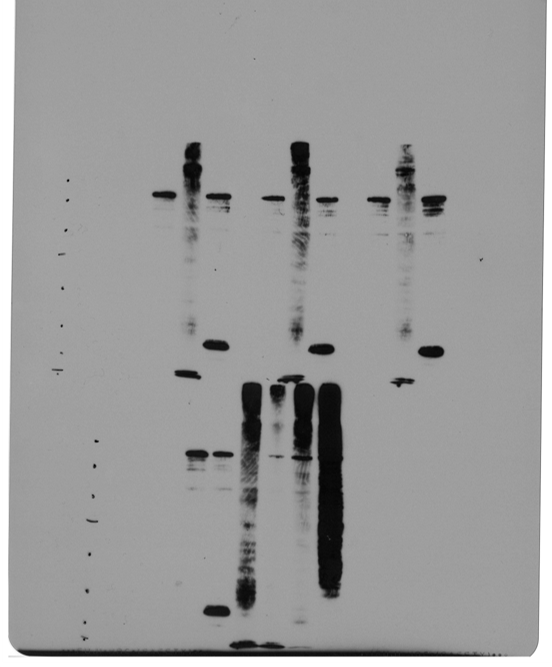

Supplement: Figure 6—figure supplement 4—source data 2. [file elife-96414-fig6-figsupp4-data2.zip › Figure 6 - figure supplement 4 - source data 2/Fig6_Supp4B_PepA_HA.tif]

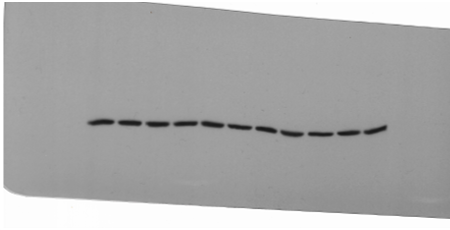

Supplement: Figure 6—figure supplement 4—source data 2. [file elife-96414-fig6-figsupp4-data2.zip › Figure 6 - figure supplement 4 - source data 2/Fig6_Supp4B_PMSF-ZVAD-Dyno-PIC_Actin.tif]

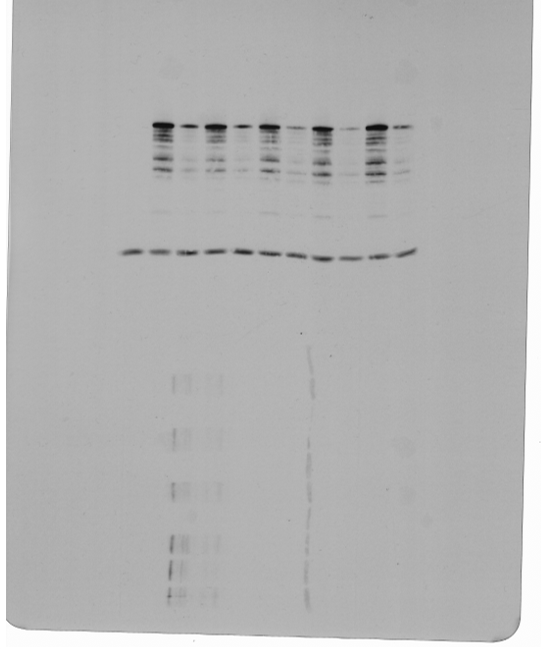

Supplement: Figure 6—figure supplement 4—source data 2. [file elife-96414-fig6-figsupp4-data2.zip › Figure 6 - figure supplement 4 - source data 2/Fig6_Supp4B_PMSF-ZVAD-Dyno-PIC_Flag.tif]

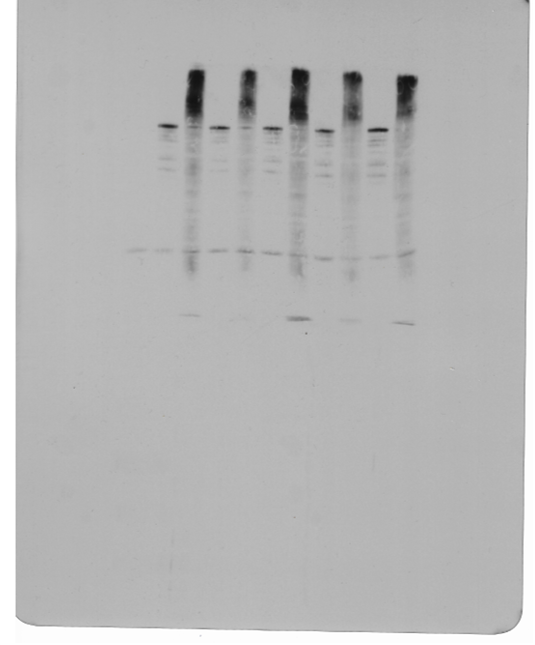

Supplement: Figure 6—figure supplement 4—source data 2. [file elife-96414-fig6-figsupp4-data2.zip › Figure 6 - figure supplement 4 - source data 2/Fig6_Supp4B_PMSF-ZVAD-Dyno-PIC_HA.tif]

Figure 7B

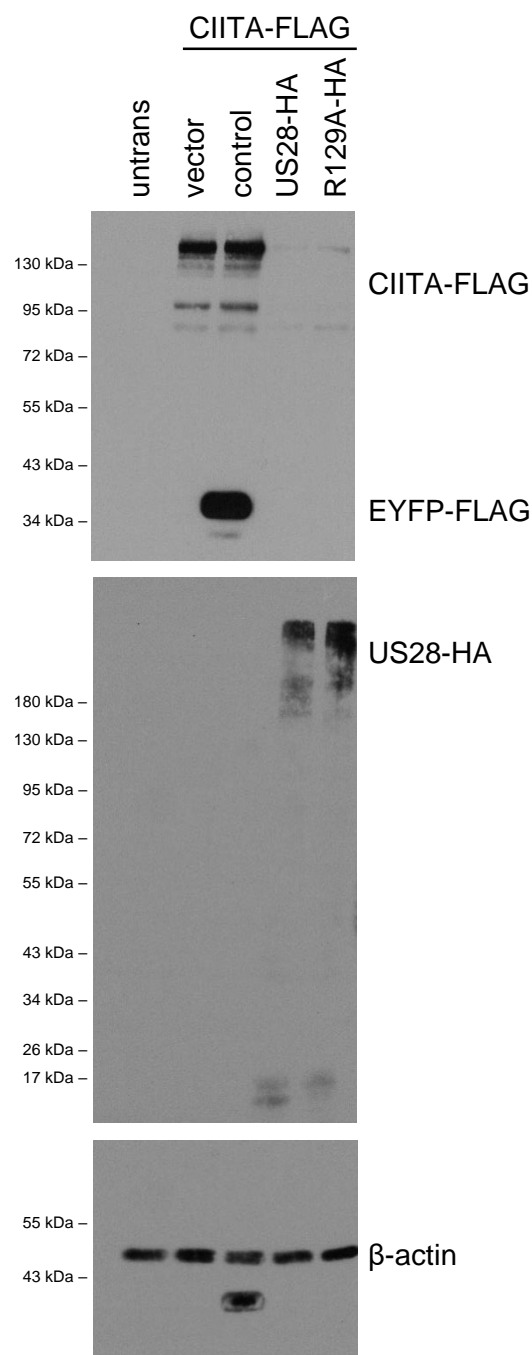

Supplement: Figure 7—source data 1. [file elife-96414-fig7-data1.zip › Figure 7 - source data 1/Fig.7B.pdf]

Figure 7C

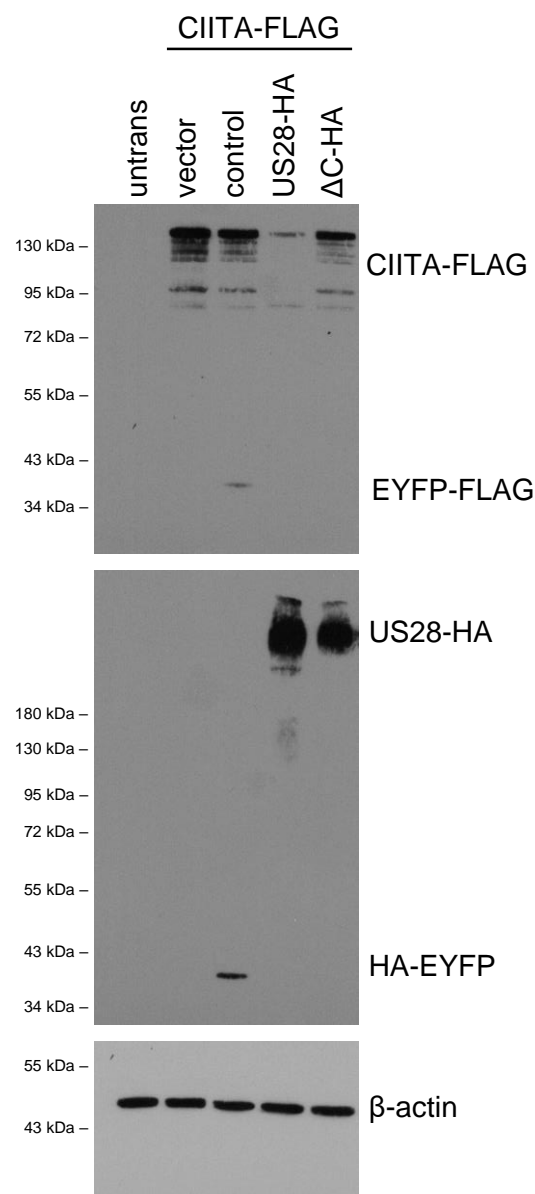

Supplement: Figure 7—source data 1. [file elife-96414-fig7-data1.zip › Figure 7 - source data 1/Fig.7C.pdf]

Figure 7H

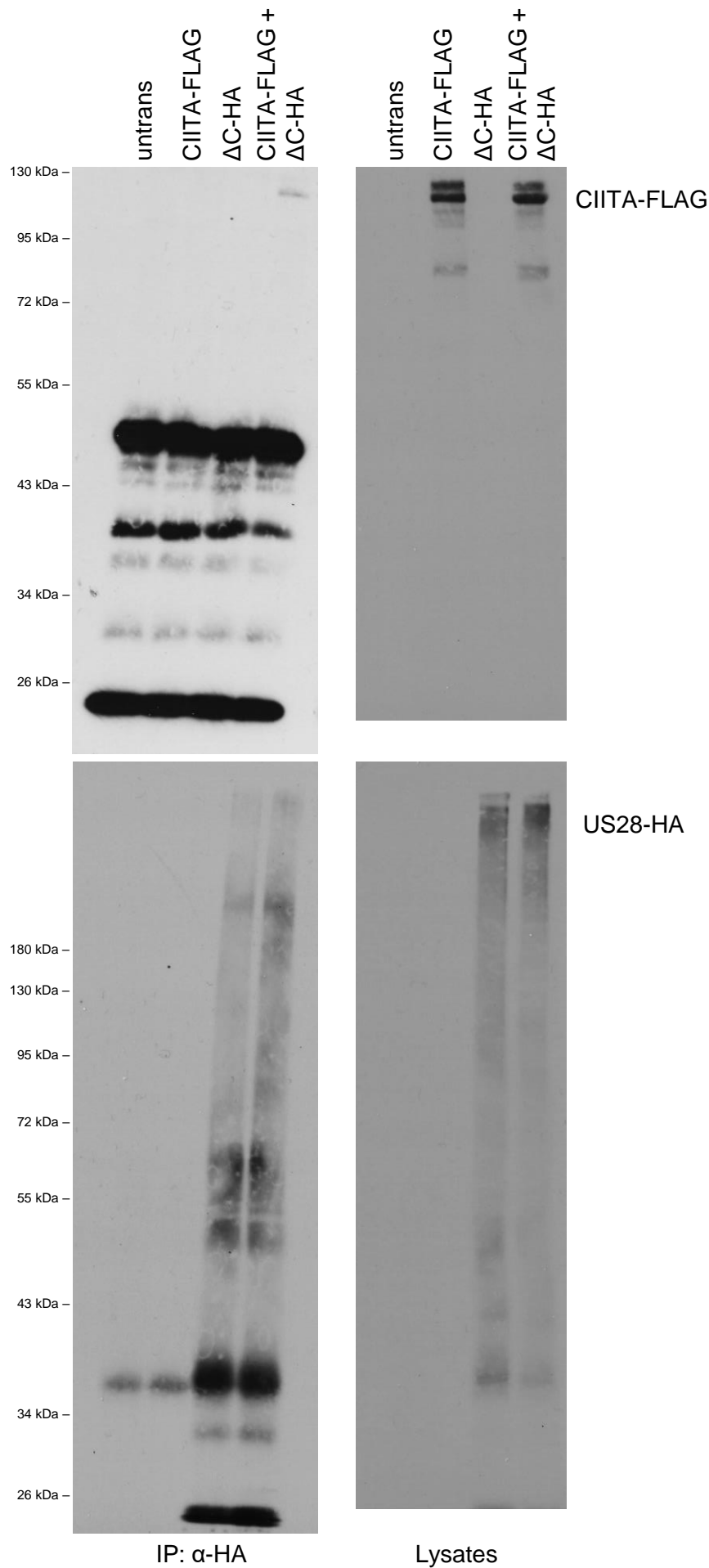

Supplement: Figure 7—source data 1. [file elife-96414-fig7-data1.zip › Figure 7 - source data 1/Fig.7H.pdf]

Figure 7I

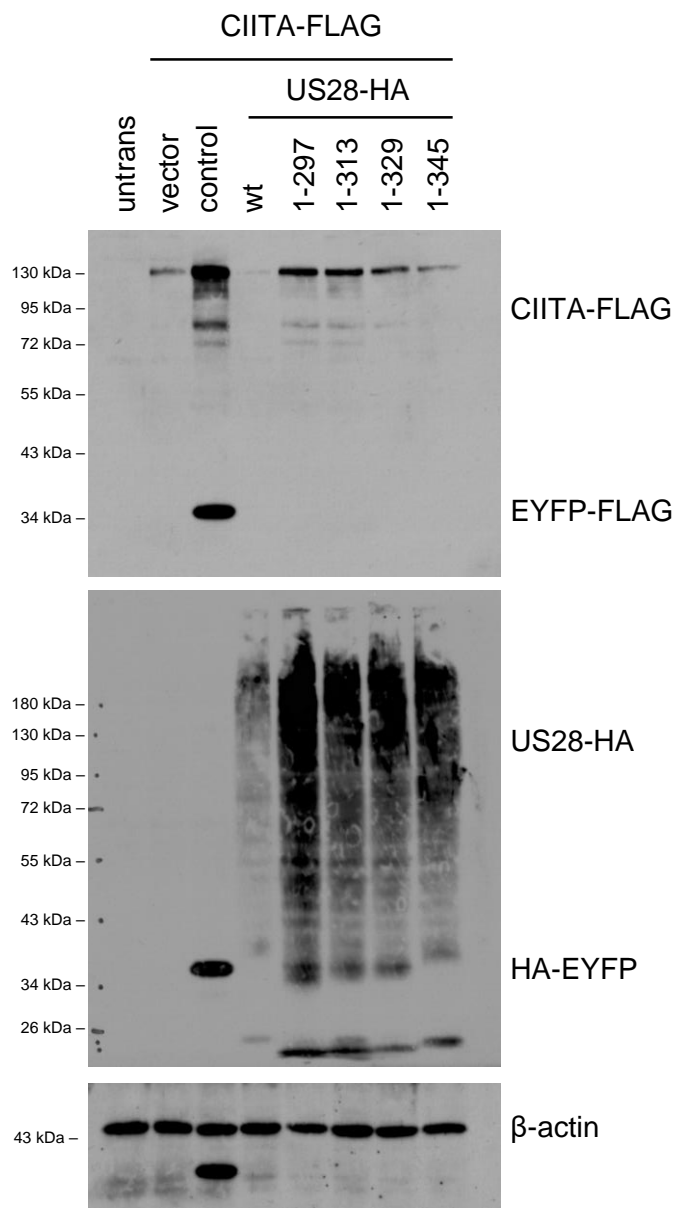

Supplement: Figure 7—source data 1. [file elife-96414-fig7-data1.zip › Figure 7 - source data 1/Fig.7I.pdf]

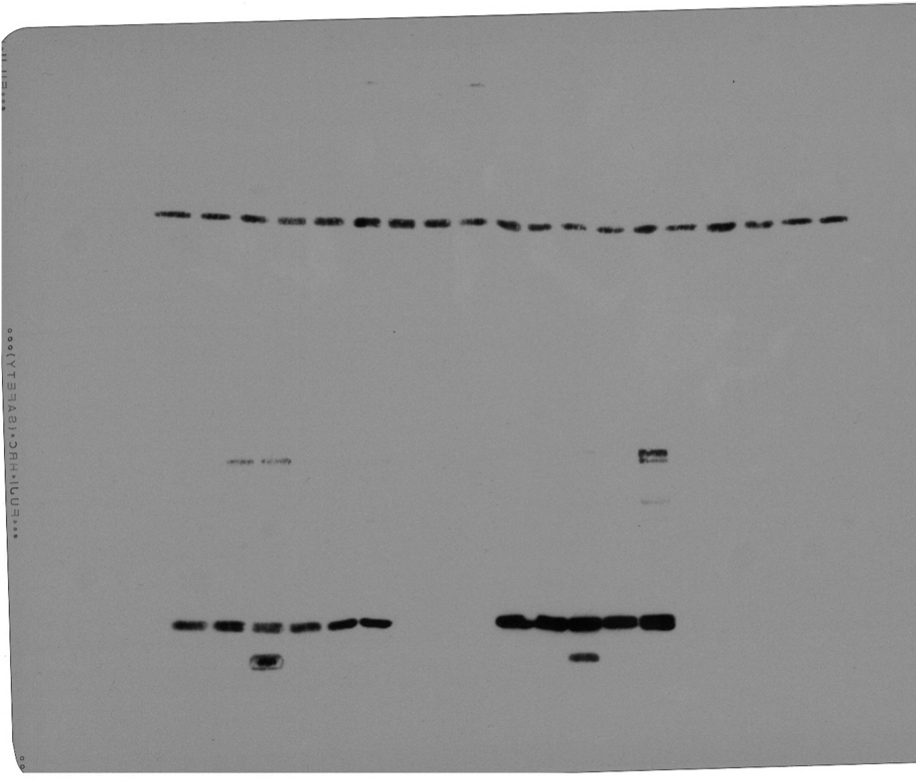

Supplement: Figure 7—source data 2. [file elife-96414-fig7-data2.zip › Figure 7 - source data 2/Fig7B_Actin.tif]

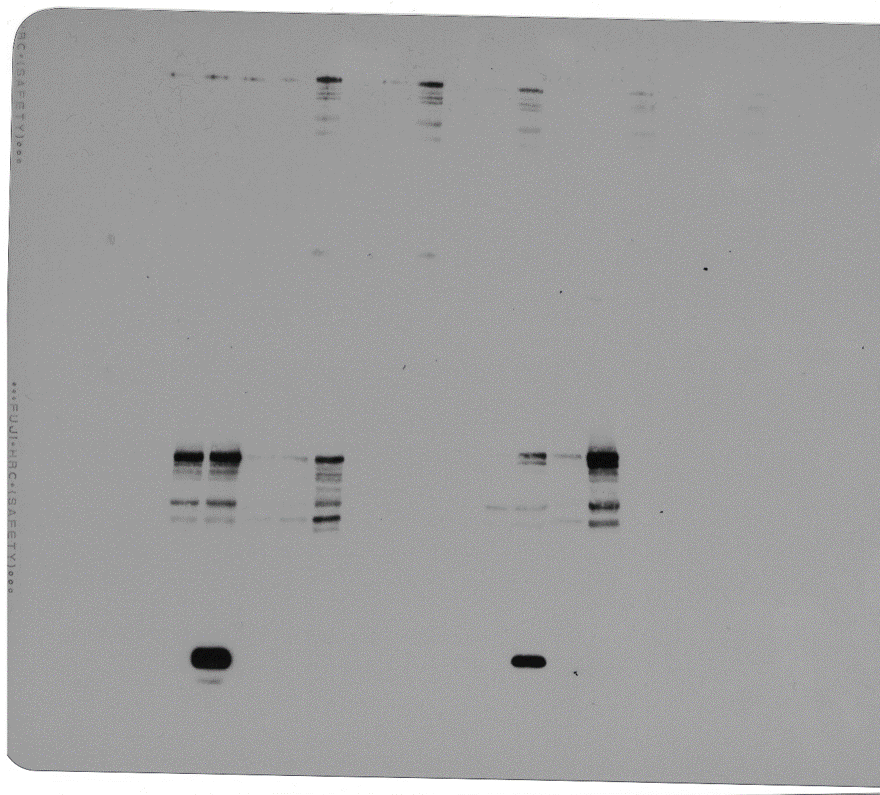

Supplement: Figure 7—source data 2. [file elife-96414-fig7-data2.zip › Figure 7 - source data 2/Fig7B_Flag.tif]

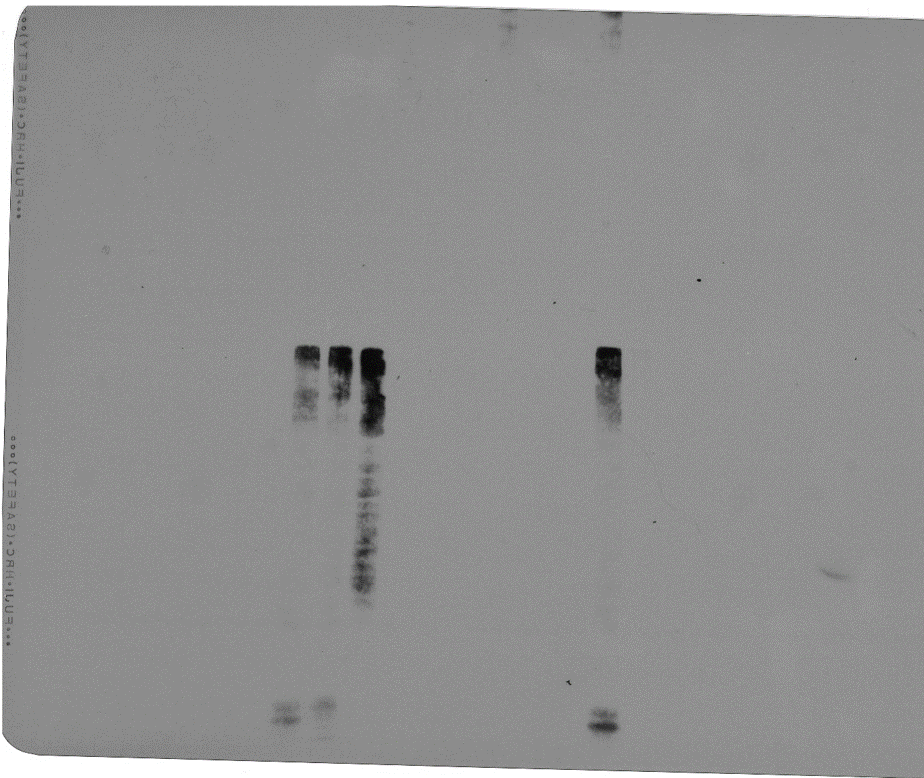

Supplement: Figure 7—source data 2. [file elife-96414-fig7-data2.zip › Figure 7 - source data 2/Fig7B_HA.tif]

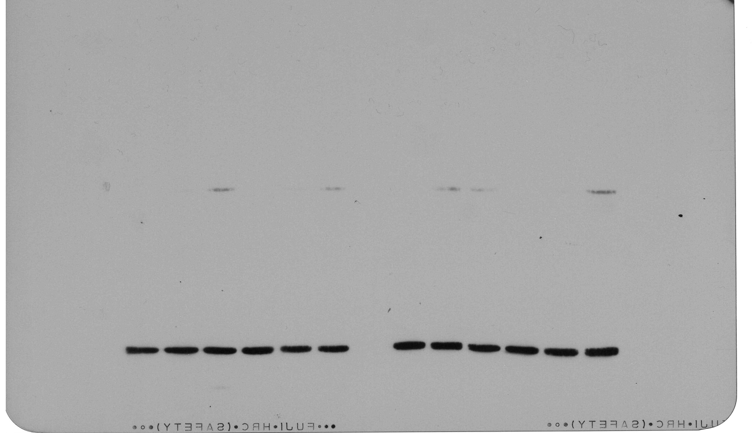

Supplement: Figure 7—source data 2. [file elife-96414-fig7-data2.zip › Figure 7 - source data 2/Fig7C_Actin.tif]

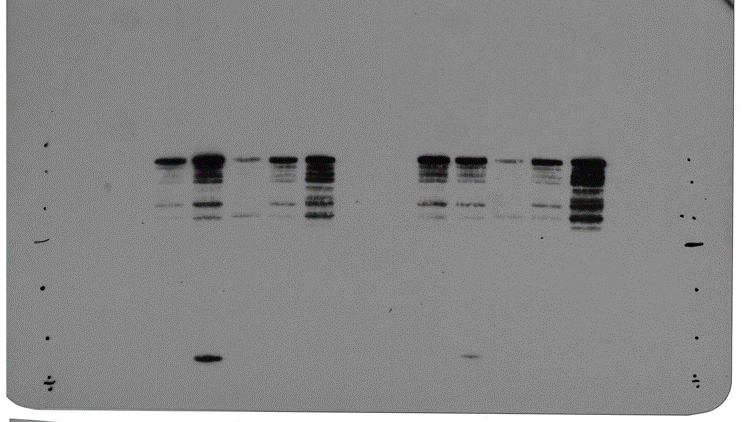

Supplement: Figure 7—source data 2. [file elife-96414-fig7-data2.zip › Figure 7 - source data 2/Fig7C_Flag.tif]

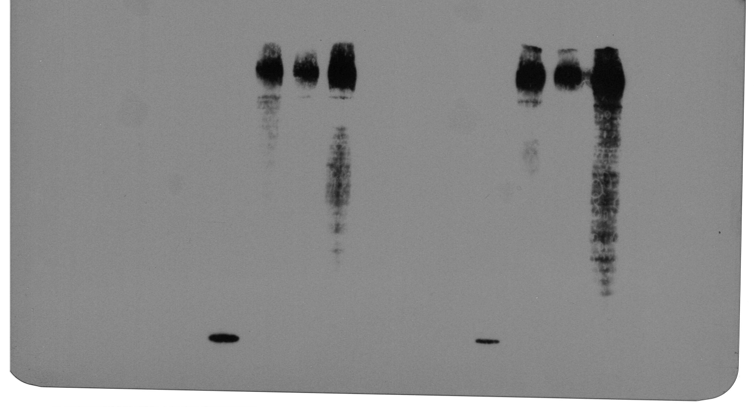

Supplement: Figure 7—source data 2. [file elife-96414-fig7-data2.zip › Figure 7 - source data 2/Fig7C_HA.tif]

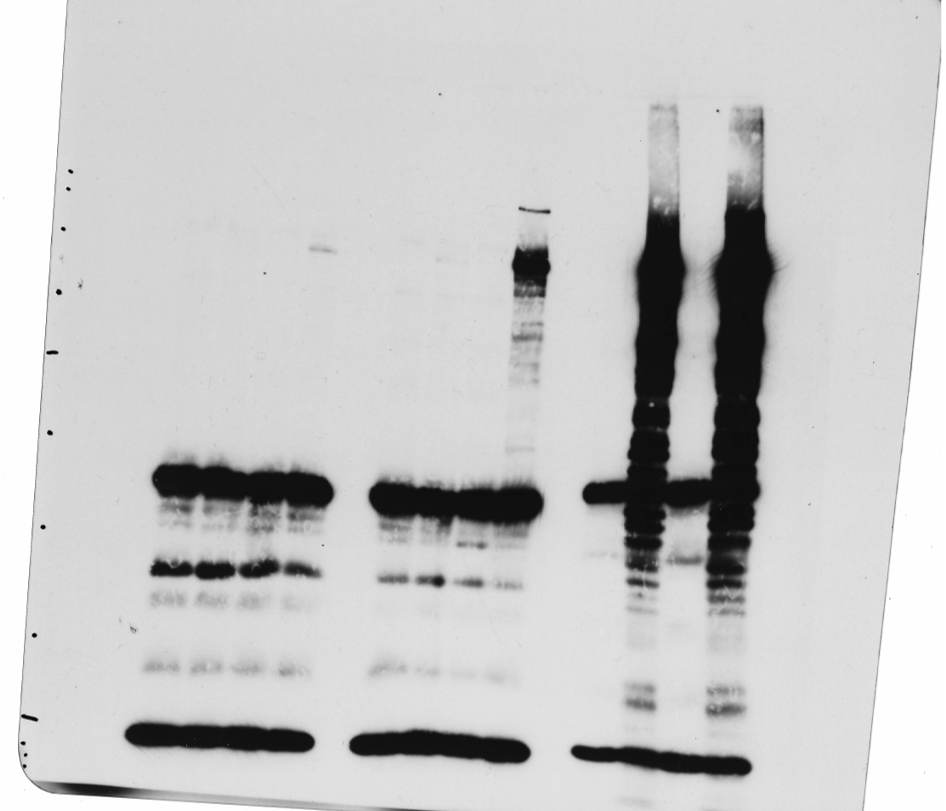

Supplement: Figure 7—source data 2. [file elife-96414-fig7-data2.zip › Figure 7 - source data 2/Fig7H_IP_Flag.tif]

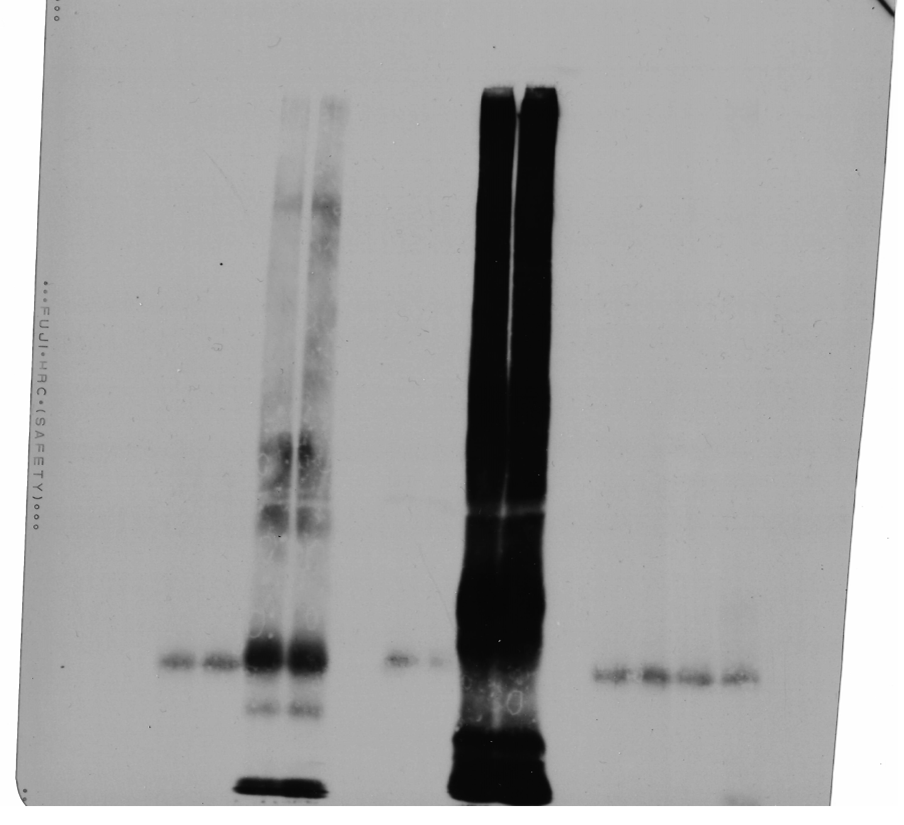

Supplement: Figure 7—source data 2. [file elife-96414-fig7-data2.zip › Figure 7 - source data 2/Fig7H_IP_HA.tif]

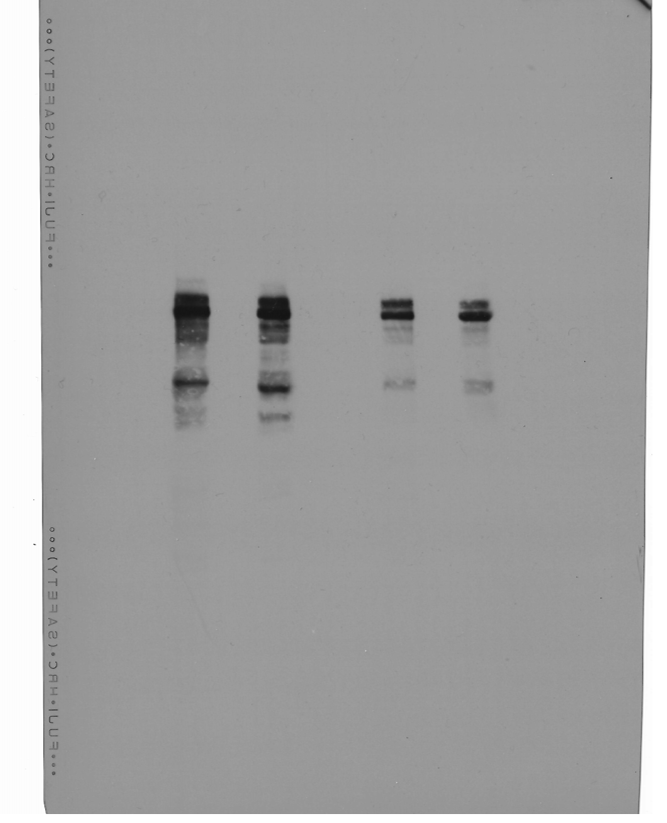

Supplement: Figure 7—source data 2. [file elife-96414-fig7-data2.zip › Figure 7 - source data 2/Fig7H_lysates_Flag_2.tif]

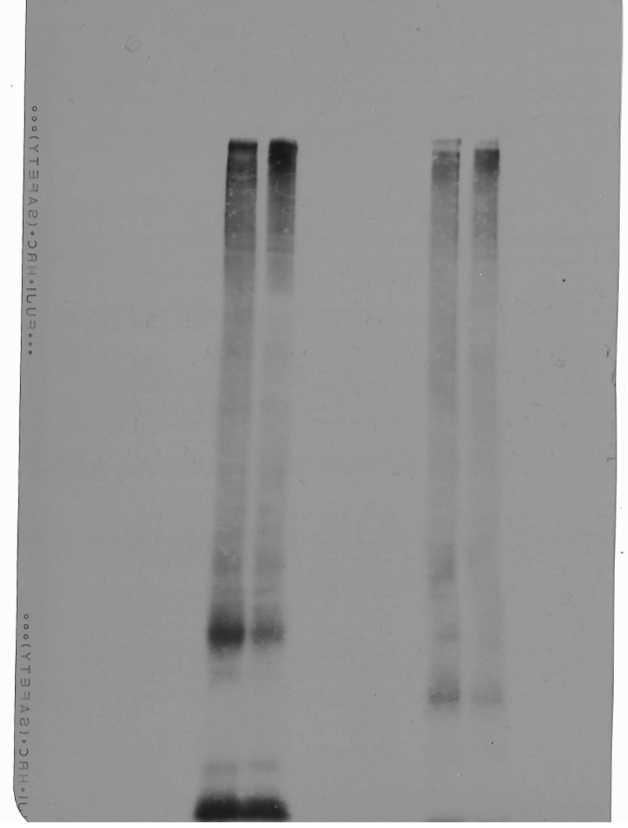

Supplement: Figure 7—source data 2. [file elife-96414-fig7-data2.zip › Figure 7 - source data 2/Fig7H_lysates_HA_2.tif]

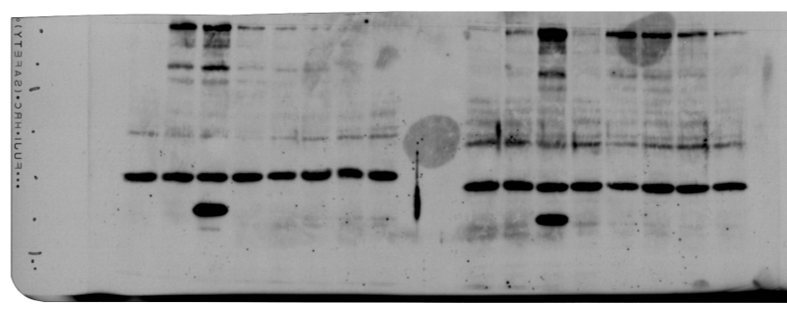

Supplement: Figure 7—source data 2. [file elife-96414-fig7-data2.zip › Figure 7 - source data 2/Fig7I_Actin.tif]

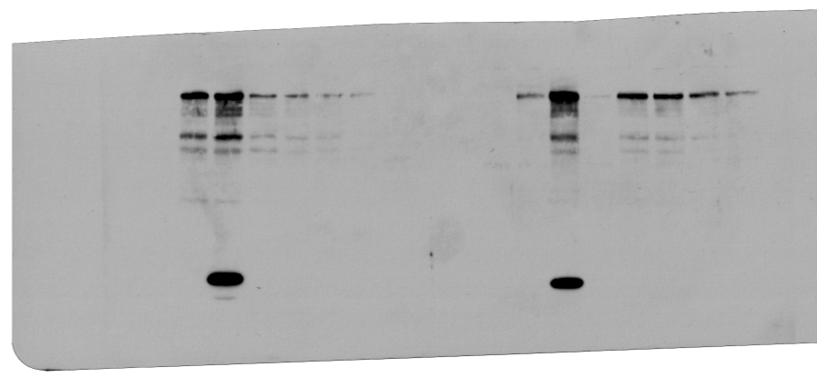

Supplement: Figure 7—source data 2. [file elife-96414-fig7-data2.zip › Figure 7 - source data 2/Fig7I_Flag.tif]

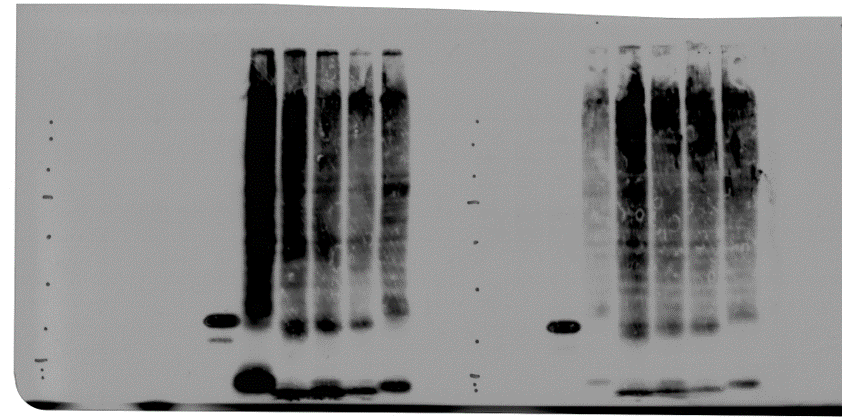

Supplement: Figure 7—source data 2. [file elife-96414-fig7-data2.zip › Figure 7 - source data 2/Fig7I_HA.tif]
